# What Makes a Branched Aromatic Compound a Crystallization Chaperone? Insights from a Comparison of Three Organic Scaffolds

**DOI:** 10.1002/chem.202501795

**Published:** 2025-08-14

**Authors:** Jan Hartenfels, Tim Berking, Ruben Pereira Rebelo, Katerina Tsimopoulou, Stefanie Schiele, Leon Stark, Wolfgang Frey, Clemens Richert

**Affiliations:** ^1^ Institute of Organic Chemistry University of Stuttgart 70569 Stuttgart Germany

**Keywords:** adamantanes, crystallization chaperone, rotational barriers, x‐ray diffraction

## Abstract

Some tetraaryladamantane (TAA) octa‐ and tetraethers have the ability to crystallize into well‐ordered lattices without full desolvation. In many cases, the solvates then yield high‐resolution X‐ray crystal structures of the encapsulated liquids. To shed light on this unusual effect of TAAs as crystallization chaperones, we have synthesized a series of spirobiflourene and porphyrin derivatives with four phenyl arms also found in TAA chaperones. Despite the structural similarity, neither of the non‐TAA compounds showed promising crystallization properties. Six new X‐ray crystal structures were obtained, but neither gave a high‐resolution structure of an encapsulated guest. Quantum chemical computations suggest that conformational changes have low activation barriers for the TAAs, which may help to adapt to the structures of guest molecules in tightly packed arrangements. These findings on supramolecular chemistry in the crystalline state may help to design new chaperones with improved properties.

## Graphical abstract

The ability to accommodate guest molecules of many different shapes in crystal lattices has been tested for teraaryl derivatives of porphyrin, spirobifluorene, and adamantane. The latter appears to offer the most favorable properties, which may be at least partially due to low activation barriers for the interconversion of rotamers.

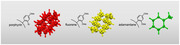

## 1 Introduction

Crystallization is one of the most fundamental processes of chemistry. It serves multiple purposes, including purification, stabilization against decomposition, and improving other properties.^[^ ^1^ ^]^ Crystals are also aesthetically pleasing. Inducing crystallization in liquids or oils can be a formidable challenge. Many compounds do not crystallize spontaneously, and many small to medium‐size molecules have a melting point below room temperature. Obtaining crystalline material for structure elucidation by X‐ray crystallography can require substantial experimental effort if neat samples are used.^[^ ^2^ ^]^

One way of overcoming the crystallization challenge for liquids is to employ chaperones. Crystallization chaperones are compounds that help to induce crystalline order in samples that resist crystallization by themselves. The concept of chaperones is established in biology,^[^ ^3^, ^4^ ^]^ but organic compounds that set up scaffolds, into which guest molecules are embedded, are also known.^[^ ^5^ ^]^ They complement the metal organic frameworks that have been used as “crystalline sponges” to soak analyte molecules into pre‐formed lattices with displacement of solvents.^[^ ^6^, ^7^ ^]^ Well known among the organic molecules that set up a scaffold upon crystallization is Dianin's compound.^[^ ^8^, ^9^, ^10^, ^11^ ^]^ This aromatic compound crystallizes into a fixed crystalline arrangement that can accommodate many different guest molecules in its central channel, but most often, the guests are disordered, and the crystals are not suitable for structure elucidation.

We have recently reported that tetraaryladamantane (TAA) ethers can crystallize in the form of solvates,^[^ ^12^ ^]^ where some of the liquid, from which the TAA was crystallized, is encapsulated in the crystal lattice in well‐ordered form.^[^ ^13^ ^]^ The first compound for which this phenomenon was observed was 1,3,5,7‐tetrakis(2,4‐dimethoxyphenyl)adamantane (TDA).^[^ ^14^ ^]^ While a number of related compounds showed no propensity to act as crystallization chaperones, 1,3,5,7‐tetrakis(2,4‐diethoxyphenyl)adamantane (TEO) did,^[^ ^15^ ^]^ and so did 1,3,5,7‐tetrakis(2‐bromo‐4‐methoxyphenyl)adamantane (TBro).^[^ ^16^ ^]^ Most recently, 1,3,5,7‐tetrakis(2‐fluoro‐4‐methoxyphenyl)adamantane (TFM) was introduced as a chaperone for larger analytes, encapsulating up to a molecular weight of 338 g/mol in resolvable fashion and up to 411 g/mol in partly disordered form.^[^ ^17^ ^]^ Well over 100 high‐resolution solvate structures with a wide range of guest compounds have been reported by us thus far, and both absolute and relative stereochemical configurations have been determined.^[^ ^18^ ^]^

**Figure 1:**
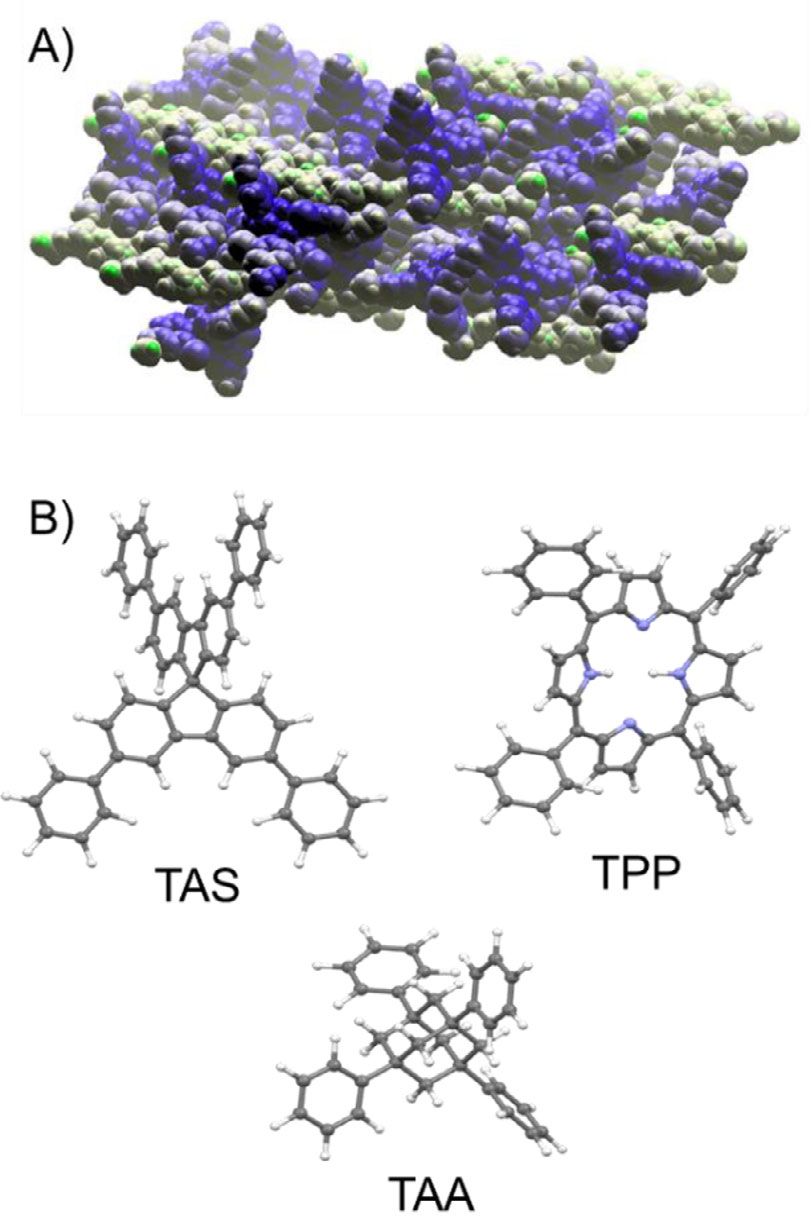
A) Close packing in the solvate structure of TBro with encapsulated farnesol.^[^ ^16^ ^]^ Molecules are shown in the space‐filling style; hydrogens were omitted for clarity. The coordinates of this structure can be found in CCDC entry No. 1970899. B) Shapes of molecular scaffold investigated in our study, as calculated using the builder of Chem3D and subsequent MM2 force field minimizations. The parent compounds of tetraphenylspirobliflourene (TAS), tetraphenylporphyrin (TPP), and tetraphenyladamantane (TAA) are shown, with unsubstituted phenyl arms.

High resolution in X‐ray structures requires closely packed, highly regular crystal arrangements, as shown in Figure 1A for farnesol, a natural product, and TBro as a chaperone. Close packing requires shape complementarity. How one scaffolding chaperone can accommodate many other molecules of varying size and shape in its crystal lattice has remained enigmatic. Shape complementarity can only exist to a certain extent for the varying guests. So far, what molecular phenomena are causing the unexpected combination of ordering and promiscuity remained unclear.

**Figure 2:**
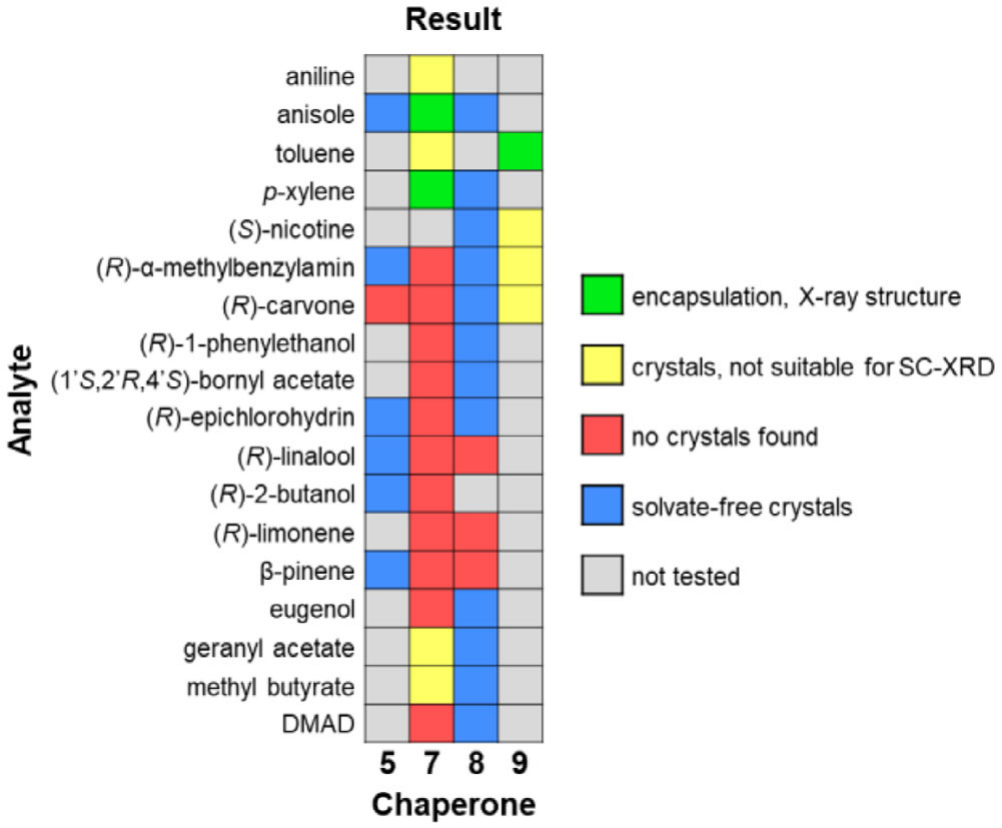
Results of crystallization experiments with spirobifluorenes as chaperone candidates and the liquids listed on the left. The thermal crystallization protocol was used, except for **8**, which was employed in diffusion‐controlled experiments, using MeOH or cyclohexane as an antisolvent.

We decided to study promiscuous encapsulation with the help of related compounds that feature the same number of distal phenyl arms as the successful TAAs named above but a different core or branching element that positions them in space. Here we report the synthesis of three spirobifluorene and 10 metallo porphyrin derivatives with phenyl substituents of the type found in TAA ethers known to act as chaperones. The shapes of the tetraphenyl parent compounds are shown in Figure 2B. As detailed below, neither of the new chaperone candidates gave an encapsulating effect of the type found for TDA and its brethren. We take these findings as an opportunity to propose that TAA ethers are structurally privileged as chaperones, combining symmetry, rigidity, and adaptability by conformational changes. We also consider activation barriers for rotation about the phenyl‐core bonds in the final part of our report.

## 2 Results and Discussion

### 2.1 Synthesis

**Scheme 1:**
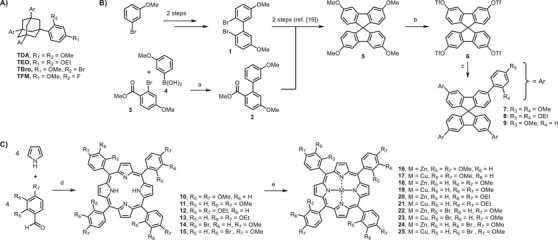
Syntheses and structures of tetraaryl chaperone candidates studied here. A) Known TAA crystallization chaperones.^[^ ^14^, ^15^, ^16^, ^17^ ^]^ B) Synthesis of spirobifluorenes. a) Pd_2_(dba)_3_, XPhos, K_2_CO_3_, PhMe, H_2_O, 80 °C, 97%; b) 1. BBr_3_, DCM; 2. Tf_2_O, pyridine, ‐20 °C to r.t.; 50% over 2 steps; c) **7**: 2,4‐dimethoxyphenylboronic acid, Pd_2_(dba)_3_, P(Cy)_3_, K_2_CO_3_, PhMe, EtOH, H_2_O, 80 °C, 55%; **8**: 2,4‐diethoxyphenylboronic acid, Pd_2_(dba)_3_, XPhos, K_2_CO_3_, PhMe, H_2_O, 80 °C, 91%; **9**: 4‐methoxyphenylboronic acid, Pd_2_(dba)_3_, P(Cy)_3_, K_2_CO_3_, PhMe, EtOH, H_2_O, 80 °C, 94%; C) Synthetic route to TPPs. d) Propionic acid, reflux, 8–23%; e) M(OAc)_2_, CHCl_3_, reflux, 2–24 hours, 40%‐quant.; M = Zn, Cu.

Scheme 1 shows the chaperone candidates synthesized for our study, together with the structure of the TAA reference compounds in part A). One framework chosen was 9,9′‐spirobifluorene. It was chosen because the spiro connectivity of the two fluorene moieties makes it rigid. It allows for placing four phenyl substituents in roughly tetrahedral geometry at the 3,3′,6,6′‐positions of the core. Assuming that the difference in size is of little functional consequence, it was not unreasonable to assume that it may give crystallization properties similar to those of TAAs.

Following a known route for the synthesis of substituted spirobifluorenes,^[^ ^19^ ^]^ 3‐bromoanisole was converted to biphenyl diether **1**, which was reacted with methyl ester **2**, accessible from **3** and **4** via Suzuki coupling. After double ring‐closing substitution to 3,3′,6,6′‐tetramethoxy‐9,9′‐spirobifluorene (**5**), the core of the scaffold was obtained. This tetraether was used as a precursor for tetratriflate **6**, but its ability to crystallize from different liquids was also tested. Thermal crystallization from anisole, β‐pinene, (*S*)‐α‐methylbenzylamine, (*R*)‐epichlorohydrin, and (*R*)‐2‐butanol gave crystalline material. Crystallographic analyses revealed that all of them were the same, solvate‐free form (monoclinic P2_1_/c) of **5** alone. Its X‐ray crystal structure is shown in Figure S19 of the Supporting Information. Crystallization attempts from (*R*)‐carvone and 12‐crown‐4 were unsuccessful, producing amorphous solids only.

Upon ether cleavage and conversion to tetratriflate **6**, a synthetic branching point was reached, from which the different target molecules were accessible via Suzuki‐Miyaura coupling. One target molecule was tetraarylspirobifluorene analog of TDA (compound **7**, Scheme 1). In this case, fourfold cross‐coupling gave the octaether in 55% isolated yield. The TEO analog, octaethylether **8,** was obtained in an even higher yield (91%), and the collection of spirobifluorenes was complemented by preparing tetramethylether **9**, again in a more than satisfactory yield of 94% for the four‐fold Suzuki coupling. Protocols and spectroscopic data for all new compounds are provided in the Supporting Information.

The target molecules of the tetraarylporphyrin series are shown in part C of Scheme 1. A total of 16 analogs of TDA, TEO, and TBro were accessed synthetically. This included the ocatmethylethers **10** and **11**, which mimic TDA itself, and an isomer with *meta*‐ and *para*‐substituted phenyl groups. The latter was chosen to reduce the steric hindrance imposed by *ortho* substituents. Likewise, TEO analogs **12** and **13** were included in the series to have compounds with the exact substituent pattern as well as its less sterically crowded isomer. The porphyrins envisioned to mimic TBro are **14** and **15**, again as the 2,4‐disubstituted and the 3,4‐disubstituted versions, respectively. Because metal complexes, rather than free porphyrins, had been described in the literature as readily encapsulating upon crystallization,^[^ ^20^, ^21^, ^22^ ^]^ both copper and zinc metalloporphyrins were synthesized (**16**–**24**, Scheme 1).

The syntheses of the porphyrins followed established protocols.^[^ ^23^, ^24^ ^]^ A variant of the Rothemund protocol was employed to induce the eight‐component macrocyclization with subsequent oxidation.^[^ ^25^ ^]^ Yields of this synthetic step, which started with pyrrole and the aldehyde of the desired phenyl groups, ranged from 8% to 18%, with the highest value for **10** and the lowest for **15**. Conversion to the metal complexes was induced by heating the free base form of porphyrins with the metal acetates in chloroform in a slight modification of a literature procedure.^[^ ^26^ ^]^ For **12**, the copper and zinc metalloporphyrins were not pursued, as the compound itself did not crystallize readily. All other metalates were obtained in yields ≥ 40% and in sufficient quantities to allow for crystallization studies.

### 2.2 Crystallization

With these compounds in hand, we proceeded to test the crystallization properties of the chaperone candidates. After an initial test for solubility in CDCl_3_, thermal crystallization experiments were performed with different liquids. Those liquids covered a certain breadth of shapes, sizes and polarities, and included chiral analytes that had previously been studied by crystallization with TAA chaperones.^[^ ^16^, ^17^ ^]^ In a typical experiment, a few milligrams of the candidate were mixed with 10–30 µL of the respective liquid in a glass vial, and the vial was placed on a hotplate set to approx. 140 °C. The mixture was heated until boiling set in or a homogeneous solution was observed. The heating was then switched off, and the set‐up was allowed to cool to room temperature with the vial remaining on the plate. The cold mixture was then inspected for crystals suitable for single crystal X‐ray diffraction (SC‐XRD).

Figure 2 gives a graphical overview of results obtained with spirobifluorenes. Compared to **5** and **6**, spirobifluorenes **7** and **9** showed lower solubility in apolar and protic organic solvents. Thermal crystallization attempts rarely resulted in crystals, and if they did, crystallization was often not reproducible. In most experiments, amorphous solids or very small needles, unsuitable for SC‐XRD, resulted from the thermal protocol. One solvate structure was obtained early on, though. This was for **9** crystallized from toluene (Figure 5 and Table 1). In the unit cell, one toluene molecule was found as a guest, disordered, with an inversion center. Even with this simple “analyte,” the solvate was considered unsuitable for structure elucidation, with too little order imposed on the guest molecule by **9**. Many other crystallization attempts with **9** also proved unsuccessful, making it an unlikely chaperone candidate.

**Table 1:** Structural data for crystal structures of chaperone candidates studied here and the corresponding data for TAA structures.

Host	Analyte	Space group	Crystal system	Unit cell volume [Å]	Density [g/cm^3^]	Mol. ratio [guest/host]	R1/wR2 [%]	Ref.
**5**	‐	monoclinic	*P* 2_1_/c	2190	1.324	‐	4.1/9.58	
TDA	toluene	monoclinic	*P* 2_1_/c	7528	1.283	1:2	5.0/8.31	[13]
TEO	toluene	triclinic	*P* $\bar{1}$	2568	1.145	1:1	5.0/9.21	[13]
**9**	toluene	orthorhombic	*P* ccn	4467	1.239	1:1	5.3/11.09	
**14**	toluene	monoclinic	*P* 2_1_/c	2701	1.516	3:1	5.5/12.83	
TDA	*p*‐xylene	monoclinic	*P* 2_1_/c	7635	1.277	2:1	6.2/7.04	[15]
TEO	*p*‐xylene	monoclinic	*C* 2/c	13667	1.132	4:1	4.4/11.49	[13]
**7**	*p*‐xylene	triclinic	*P* $\bar{1}$	2771	1.223	1:1	5.6/11.65	
TDA	anisole	monoclinic	*P* 2_1_/c	7586	1.287	1:1	4.4/10.33	[14]
**7**	anisole	triclinic	*P* $\bar{1}$	2784	1.220	1:2	6.9/16.21	
TEO	(*R*)‐α‐methylbenzamine	triclinic	*P* 1	2609	1.164	2:2	3.5/8.60	[13]
**24**	(*R*)‐α‐methylbenzamine	triclinic	*P* 1	2925	1.471	4:2	7.2/16.45	
**8**	‐	monoclinic	*P* 2_1_/c	5465	1.183	‐	15.5/34.67	

Crystallization experiments with octamethylether **7** were moderately successful in terms of encapsulating liquids. Solvate crystals were reproducibly formed from anisole and *p*‐xylene as liquids, though. Incorporation occurred at a molar ratio of 2:3 (host:guest). In the crystal structures, the spirobifluorene appeared well resolved, while the guest molecules were partly ordered or disordered (Figure 4). Very small, needle‐shaped crystals were obtained when crystallizing **7** from geranyl acetate, methyl butyrate, and (*R*)‐α‐methylbenzylamine, but their shape made them unsuitable for SC‐XRD analysis.

Thermal crystallizations from eugenol, DMAD, (*R*)‐1‐phenylethanol, β‐pinene, (*S*)‐α‐methylbenzylamine, (*R*)‐epichlorohydrin, (*R*)‐2‐butanol, (*R*)‐carvone, (*R*)‐linalool, and (*R*)‐limonene resulted in amorphous solids. So, only mono‐ or disubstituted benzene derivatives were successfully encapsulated in the crystal lattices set up by **7**. On the other hand, no solvate‐free form of octamethylether **7** was found in any of the experiments, in sharp contrast to the TAA chaperones, all of which can assemble into tightly packed, solvate‐free crystals in cases where no encapsulation is observed.

As an alternative method, diffusion‐controlled crystallization was attempted. Diffusion was induced by placing a solution of **7** in anisole in a vial containing methanol as an antisolvent, slowly lowering the solubility of the host. After 24 hours, crystals had formed that were washed with cyclohexane, dried, and the inclusion of anisole was confirmed by dissolving those crystals and measuring ^1^H‐NMR spectra. Diffusion resulted in larger crystals, but when the same approach was used for (*R*)‐carvone and eugenol, the same small needles as by the thermal method were obtained. For the latter liquid, a crystal was found suitable for SC‐XRD. Here, strongly disordered methanol molecules had been encapsulated by **7**, rather than the natural product (Figure S20, Supporting Information).

Crystallization runs with octaethylether **8** gave larger crystals with the diffusion‐controlled method than from thermal crystallizations, but the latter again gave mostly small, needle‐shaped crystals deemed unsuitable for SC‐XRD. In all successful crystallization runs, the same solvate‐free form of the tetraarylspirobifluorene was obtained. This crystal structure of **8** is shown in Figure S21 of the Supporting Information. In this case, a tight packing of the scaffolding molecule alone is observed, with ethoxy groups reaching into the space between aryl moieties, reminiscent of the packing of the *n*‐propyl octaether of TAA, for which no encapsulation was reported.^[^ ^15^ ^]^ Apparently, here, the solvate‐free crystal system is energetically or kinetically much preferred over solvates, as neither anisole, *p*‐xylene, α‐(*R*)‐methylbenzylamine, eugenol, (*R*)‐1‐phenylethanol, nicotine, geranyl acetate, bornyl acetate, methyl butyrate, dimethyl acetylenedicarboxylate, (*R*)‐carvone, or (*R*)‐epichlorohydrine were encapsulated. Amorphous solids were observed in attempts to crystallize **8** from β‐pinene, (*R*)‐linalool, and (*R*)‐limonene, as compiled graphically in Figure 2.

**Figure 3:**
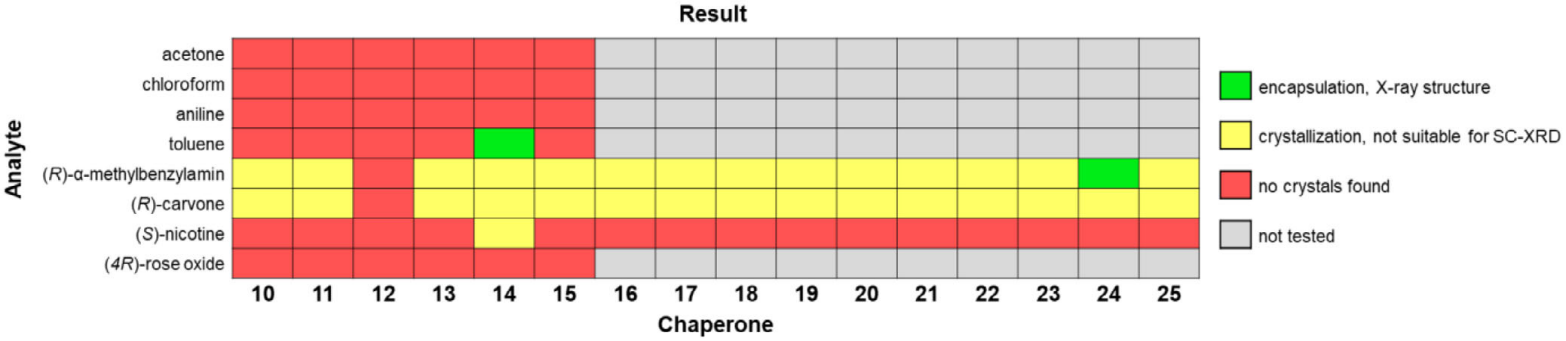
Graphical compilation of the results of crystallization attempts with tetraarylporphyrins and liquid analytes or solvents. Crystallization attempts from acetone and chloroform were done by evaporation of the solvent.

**Figure 4:**
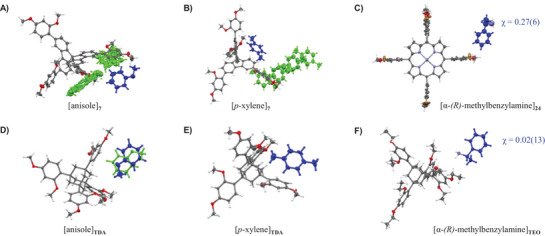
Comparison of solvate structures of non‐TAA and TAA chaperones. Excerpts of the unit cells with one chaperone molecule and the best‐resolved guest molecule are shown as ORTEP representations at 50% probability level. When electron density for more than one position of the guest was found, blue and green structures are shown, each representing a fraction of that electron density. The upper row (A‐C) shows solvate structures with **7** and **24** as chaperone candidates, and the lower row (D‐F) shows the corresponding structures with TAA chaperones for comparison. The chaperones are indicated as subscripts after the brackets denoting the guest. For the chiral guests of C) and F), the Flack parameter χ is given in blue, next to the analyte. The structures in the lower row were generated from CCDC entries 1483172, 1978501 and 1970890, respectively. Colors: C, gray; H, white; O, red; N, blue; Br, orange; Zn, indigo. See Table 1 for additional data.

Crystallization with porphyrins was complicated by the deep color of the tetrapyrroles. Even in small vials, it was difficult to discern whether the solid had fully dissolved in the hot liquid when attempting to use the thermal crystallization protocol. If crystallization did set in, it was difficult to pick suitable crystals from a mother liquor too dark to grant full visual access. Many crystallization runs resulted in either amorphous solids or crystals that were unsuitable for analysis via SC‐XRD. Figure 3 gives an overview of the results of the crystallographic screen with this class of scaffolds. Briefly, thermal crystallizations of (metallo)TPPs with both α‐(*R*)‐methylbenzylamine and (*R*)‐carvone invariably resulted in low‐quality crystals. One suitable single crystal of **24**/α‐(*R*)‐methylbenzylamine was obtained, and an excerpt of the unit cell is shown in Figure 4c. The full asymmetric unit is depicted in Figure S22 of the Supporting Information. The excerpt shows the most well‐resolved analyte molecule of the unit cell and only one of multiple domains containing it, including conformations in which the analyte is coordinated to the metal center of the TPP. Even for this molecule, the high Flack parameter makes it difficult to determine an absolute configuration. In contrast, the TEO structure of α‐(*R*)‐methylbenzylamine determined earlier,^[^ ^16^ ^]^ and shown in Figure 4F contains a well‐ordered analyte, with a Flack parameter well below the recommended value of ≤ 0.10.

**Figure 5:**
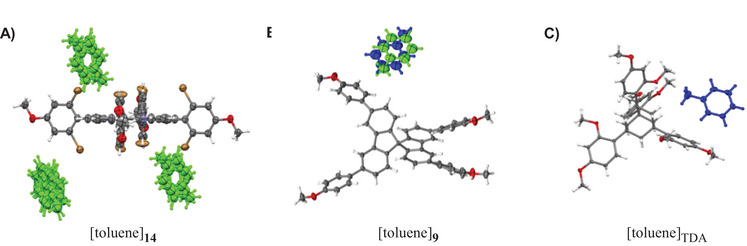
Comparison of the X‐ray crystal structures of toluene solvates for two non‐TAA and one TAA chaperone. A) Porphyrin chaperone **14**, B) spirobifluorene chaperone **9**, and C) tetraaryladamantane TDA. The guest molecules are shown in blue (and additionally in green, if more than one position is partially occupied). Note that in A) both the host and the guest molecules are disordered, so that it appears as if eight bromine substituents were present, even though just four of them are occupying two alternative positions with 50% occupancy. Also, in B) two toluene orientations were found, related to each other by an inversion center. The well‐ordered TAA crystal structure of C) was drawn from CCDC entry 1918380. Depicted are ORTEP representations at the 50% probability level with the following colors: C, grey; H, white; O, red; N, blue; Br, orange. See Table 1 for crystallographic data and stoichiometries.

For the TPPs without a metal center, only **14** gave a solvate crystal suitable for X‐ray crystallography. This was the toluene solvate shown in Figure 5A. Interestingly, the structure showed both conformational disorder for the porphyrin host, with bromophenyl moieties rotated 180 ° relative to each other, and positional disorder for the guest. Three toluene molecules per porphyrin were detected, and either of the three solvent domains showed strong disorder. As Figure 5 shows, the level of disorder was greater than for the corresponding toluene solvate of spirobifluorene **9**, where only two positions for the guest were detected, related by an inversion center and resolvable by crystallographic analysis. Again, the comparison with the corresponding TAA solvate (TDA, Figure 5C) shows clearly how much better suited the latter is as a chaperone for structure elucidation.

**Figure 6:**
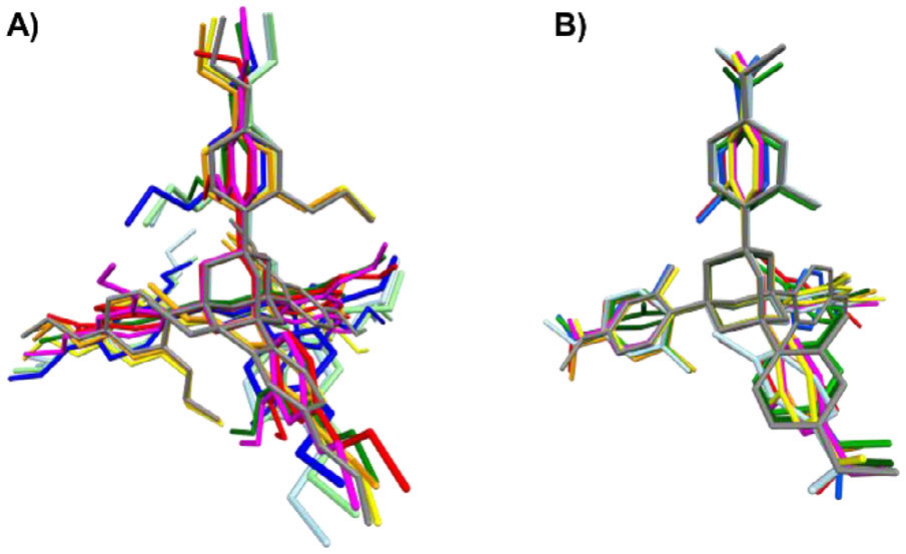
Overlay of conformations of tetraaryladamantanes in different solvate structures. A) Conformations of nine TEO conformations, as observed in X‐ray crystal structures with different liquids (CCDC entries 1970890, 1978497, 1978498, 1978499, 1918377, 1978246, 1970890, 1970894, 1970889). B) Conformations of all nine TFM solvate structures ^[^ ^17^ ^]^ currently available in the CCDC database.

The ability of the phenyl arms of TAAs to adopt different conformations in solvate crystals is also apparent from the overlay shown in Figure 6. The first overlay of conformations is for TEO (Figure 6A). There is a range of dihedral angles between phenyl and adamantane rings, and the ether groups are also adopting diverse conformations to accommodate the respective liquid. Likewise, TFM shows rotation about the bond between arm and core, and the rotatable substituent at the para position again changes its position from one structure to the next (Figure 6B). We obtained too few X‐ray crystal structures of porphyrins and spirobifluorene to perform an analysis on the same level. However, the available data suggest much reduced conformational flexibility for the former and fewer dihedral angles between arms and core for the latter (Figure S32, Supporting Information).

Upon the suggestion of a referee, we also analyzed representative X‐ray crystal structures for close intermolecular contacts by calculating Hirshfeld surfaces and generating 2D fingerprint plots.^[^ ^27^ ^]^ This is shown in Figure S31 of the Supporting Information for structures of TEO with and without toluene, as well as spirobifluorene **9** and porphyrin **14** with the same liquid as inclusion compounds. There is no unusual level of close contacts in the crystals of the TAA, nor were there other special features in the fingerprint plots discernible to us that correlate with the ability to form well‐ordered solvate structures or not.

### 2.3 Theoretical Work

Taken together, the results from our crystallization experiments with spirobifluorenes and porphyrins indicate that they do not perform on the same level as the adamantanes. Since both of the alternative scaffolds tested were larger than the TAAs, it seemed unlikely that there was insufficient space left for encapsulation when the scaffold molecules assembled into crystal lattices. Rather, some molecular feature appears to be favoring well‐ordered solvates for the adamantanes that the other two classes of tetraaryl scaffolds do not possess. Something facilitates close packing of the chaperone against the different shapes of guest molecules that is not easy to achieve for the other structures. We suspected that one factor is the ease of rotating the phenyl arms about the aryl‐adamantane axis.

**Figure 7:**
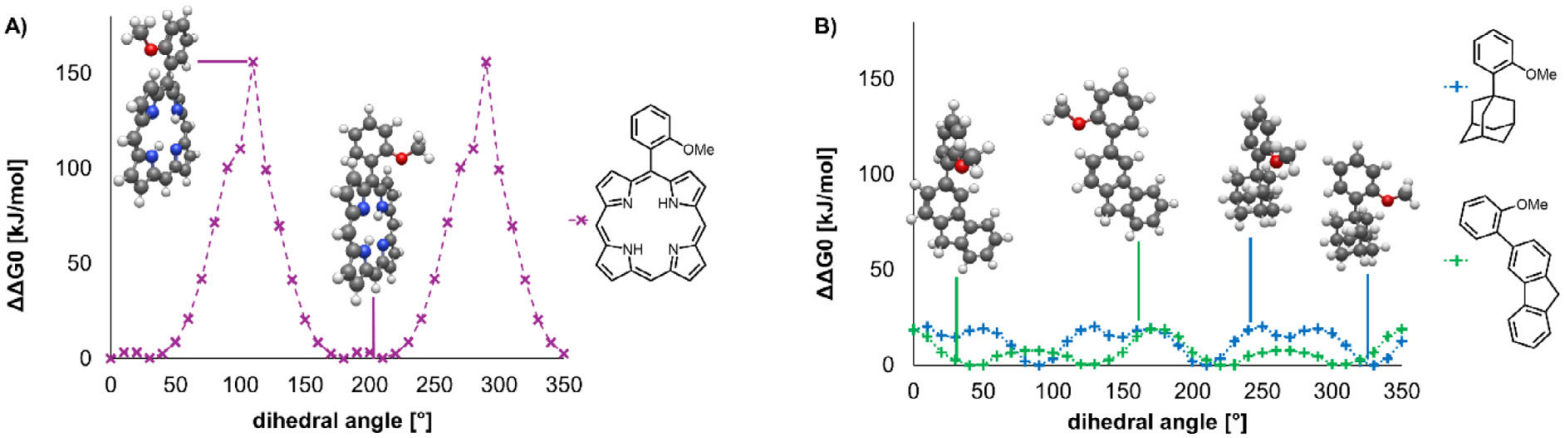
Calculated potential‐energy diagrams for the rotation about the phenyl‐core bond in three model compounds mimicking the steric situation in A) porphyrin and B) spirobifluorene and adamantane chaperone candidates. Low‐energy rotamers were identified by a global optimizer algorithm (GOAT) on the GFN2‐xTB level of theory.^[^ ^29^, ^30^ ^]^ Dihedral angles for rotation between core and *ortho*‐substituted phenyl moieties were constrained and changed in 10° steps for a total of 180° in the cases of porphyrin and fluorene or 120° in the case of adamantane. Quantum chemical computations were then performed on the B3LYP/def2‐SVP level of theory using the ORCA 5.0 software package. ^[^ ^31^, ^32^, ^33^, ^34^, ^35^, ^36^ ^]^ The plots show the energies obtained for each conformer and point‐to‐point fit lines. The structures of the highest and lowest energy conformers, as observed in these calculations, are shown above or beside a maximum or minimum. Line drawings on the right‐hand side of each plot show the covalent structure of the model compounds.

To shed light on this, we performed exploratory quantum chemical computations on the substructures of the chaperone candidates shown in Figure 7. Energies of conformers resulting from the rotation of an aryl group relative to the scaffold were calculated. Fragments with one aryl substituent were considered reasonable model compounds, assuming that rotation occurs independently for the four aryl substituents of a chaperone candidate. The computational work included the *ortho*‐methoxyphenyl fragments of porphyrin, fluorene, and adamantane. For the *ortho*‐substituted porphyrin (Figure 7A), a barrier > 150 kJ/mol was obtained, almost certainly leading to atropisomerism at room temperature, in agreement with the observation of such isomers in NMR spectra. Based on the theoretical results, a rotation about the aryl‐porphyrin axis requires deformation of the porphyrin ring due to steric conflicts. This conclusion is consistent with the findings of Okuno et al., who investigated rotational barriers for phenylporphyrins.^[^ ^28^ ^]^

For both the methoxyphenyladamantane and its fluorene analog, much lower barriers between high‐ and low‐energy conformations were suggested by the computations (Figure 7B). Numerical values, including data for two other TAA fragments, can be found in chapter 6 of the Supporting Information. The ability to rotate phenyl arms almost freely, adopting many conformations without a large energy penalty, may be one reason why TAA chaperones pack tightly around analyte molecules upon crystallization. This, together with the ability to adopt different conformations of the ether side chains, may lead to what may be called “induced shape complementarity” when a crystal lattice is set up by the chaperone in the presence of a liquid. While the rotation about the phenyl‐adamantane axes has no more than a modest activation barrier, placing the *ortho*‐substituent of a phenyl ring *trans* to one of the methylene groups of the adamantane core still leads to a local energy minimum and is preferred, even in solvate crystals. Overall, we suspect that shape, symmetry, rigidity, polarity, and conformational flexibility are key factors that determine whether a molecule acts as a crystallization chaperone or not.

## 3 Conclusions

Tetraphenyladamantane tetra‐ and octaethers show an unusual propensity to crystallize as solvates with well‐ordered solvent molecules encapsulated in the crystal lattices. We studied this phenomenon by synthesizing similar compounds with four disubstituted phenyl arms at the distal ends of rigid branching elements/cores. Neither of the new compounds had the same chaperone qualities as TDA, TEO, TBro, or TFM, even though similarities in polarity and shape were aimed for in the design process. The DFT calculations suggest that a low rotation barrier exists for the TAA octaethers, which may help to adapt to the shape of a given guest molecule when assembling into a crystal lattice. Thus, long‐range order may be achieved, even in rapid thermal crystallization processes that occur without (full) desolvation.

## Supporting Information

Materials and methods, synthetic protocols, NMR spectra, and crystallographic data are provided in the Supporting Information. Deposition numbers CCDC 2432456 (for **5**), CCDC 2432455 (for **7**/anisole), CCDC 2432453 (for **7**/p‐xylene), CCDC 2432457 (for **7**/methanol), CCDC 2432458 (for **8**), CCDC 2432450 (for **9**/toluene), CCDC 2432451 (for **14**/toluene), and CCDC 2432452 (for **24**/α‐(*R*)‐methylbenzylamine) contain the crystallographic data for the new structures reported in this paper. These data are provided free of charge by the joint Cambridge Crystallographic Data Centre and Fachinformationszentrum Karlsruhe.

## Conflict of Interest

The authors declare no conflict of interest.

## Supporting information



Supporting Information

## Data Availability

The data that support the findings of this study are available in the supplementary material of this article.
