# Supplementary material for: What Makes a Branched Aromatic Compound a Crystallization Chaperone? Insights from a Comparison of Three Organic Scaffolds
Source: Chemistry. 2025 Aug 14;31(54):e01795. doi: 10.1002/chem.202501795 (PMC12462234; doi:10.1002/chem.202501795)
Supplement: Supplementary file 1 — Supporting Information [file CHEM-31-e01795-s001.pdf]

Supporting Information

for manuscript entitled

What Makes a Branched Aromatic Compound a Crystallization Chaperone? Insights from a Comparison of Three Organic Scaffolds

by

Jan Hartenfels, Tim Berking, Ruben Pereira Rebelo, Katerina Tsimopoulou, Stefanie Schiele, Leon Stark, Wolfgang Frey and Clemens Richert

Contents

1. General Information S1
2. Synthetic Protocols S2
3. NMR Spectra S3
4. Crystallization Protocols S31
5. Additional Information on X-ray Crystal Structures S32
6. Theoretical Work S44
7. References for Supporting Information S49

1. General Information

Chemicals and solvents were purchased from Sigma–Aldrich (Darmstadt, Germany), TCI Europe (Zwijndrecht, Belgium), and Fluorochem (Dublin, Ireland) and were used without purification. Reactions were performed under ambient air, unless noted otherwise and were monitored using thin-layer chromatography using Macherey-Nagel ALUGRAM® Xtra SIL G/UV254 silica sheets. Compounds were visualized with either UV light or a phosphomolybdato cerium (IV) sulfate staining solution (25 g phosphomolybdic acid monohydrate, 10 g cerium(IV) sulfate tetrahydrate, 60 mL conc. sulfuric acid, diluted with water to 1 L). Purification of target molecules via column chromatography was done using silica from Macherey-Nagel (Düren, Germany) or SiliCycle (Québec City, Canada). NMR spectra were collected by a Bruker Avance 300 or Ascend 400 spectrometer, ^{1}H-NMR data was measured at 300 or 400 MHz and ^{13}C-NMR data at 75 or 101 MHz. Chemical shifts δ in ppm are given relative to the TMS signal in deuterated chloroform (0.00 ppm) or the residual solvent peak in other deuterated solvents. Multiplicity is reported as s, singlet; d, doublet; t, triplet; quin, quintet; m, multiplet and coupling constants J are reported in Hz. Exploratory MALDI-TOF mass spectra were recorded on Microflex spectrometer (Bruker Daltonics, Bremen, Germany), Axima Confidence (Shimadzu, Duisburg, Germany). High resolution mass spectra were recorded on a Bruker micrOTOF-Q spectrometer (ESI-HRMS). Single crystal X-ray diffraction analysis was performed using a KAPPA APEX II DUO diffractometer from Bruker AXS (Karlsruhe, Germany). Data was collected at a temperature of 150 K using either a Mo Kα (λ=0.71073 Å) or a Cu Kα (λ=1.54178 Å) source. Refinement and reduction of the data was done using the SAINT program package. Absorption correction was done with SADBAS3. SHELXS97[S1] was used to solve the structures using direct methods and SHELXL-2014/7[S2] least-squares methods were applied for refinement. Positions of hydrogen atoms were calculated using riding-hydrogen models. Crystallographic data was visualized using Mercury (version 4.0).[S3] X-ray structures shown in this publication were deposited to the Cambridge Crystallographic Data Centre (CCDC, https://www.ccdc.cam.ac.uk), CCDC numbers as well as structural data are shown below and are freely available by the joint Cambridge Crystallographic Data Centre and Fachinformationszentrum Karlsruhe at http://www.ccdc.cam.ac.uk/structures.

2. Synthetic Protocols

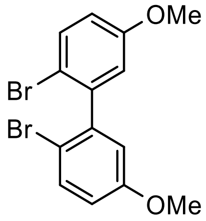

2,2'-Dibrom-5,5'-dimethoxy-[1,1'-biphenyl] (1)

The target compound was synthesized using a slight modification of a literature protocol.[S4] To a flame-dried Schlenk flask, magnesium turnings (998 mg, 41.1 mmol, 1.3 equiv) were added under a nitrogen atmosphere. A solution of dibromoethane (0.27 mL, 3.16 mmol, 0.1 equiv) and 3-bromoanisole (4.0 mL, 31.6 mmol, 1.0 equiv) in dry THF (24 mL) was added dropwise to the magnesium. The reaction mixture was refluxed for 30 min at 80 °C and then left to cool to room temperature. Any precipitate at this stage was dissolved again using gentle heating. This mixture was then added dropwise using a transfer canula to a solution containing 3-bromoanisole (4.0 mL, 31.6 mmol, 1.0 equiv) and Pd(dppp)Cl_2 (372 mg, 632 µmol, 2 mol%) in dry THF (24 mL). The reaction mixture was refluxed for 1 h at 80 °C, cooled to 0 °C, and quenched using 2M HCl (50 mL). The aqueous phase was extracted with DCM (3 × 50 mL), the combined extracts were washed with sat. $NaHCO_3$ (50 mL), dried over Na_2SO_4, filtered and solvents removed under reduced pressure. The product, 3,3’-dimethoxy-[1,1’-biphenyl], was purified via distillation and used directly in the next step. TLC (petroleum ether/dichloromethane, 2/1, *v/v*): R_f = 0.25; ^{1}H-NMR (300 MHz, $CDCl_3$): δ (ppm) = 7.35 (t, *J*=7.9 Hz, 2H), 7.12 – 7.19 (m, 2H), 7.19 – 7.25 (m, 2H), 6.90 (ddd, *J*=8.2, 2.8, 0.8 Hz, 2H), 3.87 (s, 6H).

For the bromination, again, the protocol is a slight modification of one found in the literature.[S5] In a two-necked round bottom flask, 3,3'-dimethoxy-[1,1'-biphenyl] was dissolved in acetic acid (56 mL) and bromine (3.4 mL, 66.3 mmol, 2.1 equiv) was added slowly via a dropping funnel. After complete addition, the reaction mixture was stirred at room temperature for 5 h and then quenched with a sat. Na_2SO_3 solution (50 mL). The suspension was stirred for 1 h, diluted with water (50 mL) and extracted with DCM (4 × 30 mL). The combined extracts were washed with sat. NaCl solution, dried over Na_2SO_4, filtered and the solvent removed *in vacuo*. The residue was recrystallized from ethanol to yield the brominated biphenyl **1** (7.03 g, 18.9 mmol, 60% over 2 steps) as colorless needles. TLC (petroleum ether/dichloromethane, 2/1, *v/v*): R_f = 0.10. Spectroscopic data were in agreement with the literature.[S5] ^{1}H-NMR (300 MHz, $CDCl_3$):

δ (ppm) = 7.53 (d, *J*=9.1 Hz, 2H), 6.82 (dd, *J*=3.1, 9.1 Hz, 2H), 6.79 (d, *J*=2.7 Hz, 2H), 3.81 (s, 6H).

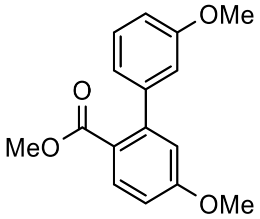

Methyl 2-(3-methoxyphenyl)-4-methoxybenzoate (2)

In a flame-dried Schlenk flask under nitrogen atmosphere, methyl benzoate **3** (1.50 g, 6.12 mmol, 1.0 equiv), boronic acid **4** (1.40 g, 9.18 mmol, 1.5 equiv), potassium carbonate (1.60 g, 12.2 mmol, 2.0 equiv), $Pd_2(dba)_3$ (56 mg, 61 µmol, 1 mol%), and XPhos (117 mg, 245 µmol, 4 mol%) were suspended in a mixture of toluene (20 mL) and water (6 mL), both purged with nitrogen, and degassed using 3 freeze-pump-thaw cycles. The reaction mixture was stirred under nitrogen atmosphere at 80 °C for 4 h until full conversion was observed via TLC. The reaction was left to cool to room temperature, diluted with DCM (90 mL) and water (50 mL) and the aqueous layer was extracted with DCM (3 × 10 mL). The combined extracts were washed with a sat. NH_4Cl solution, dried over anhydrous sodium sulfate, filtered, and the solvent removed under reduced pressure. The resulting oil was purified via column chromatography (silica, petroleum ether/ethyl acetate, 9/1, *v*/*v*) and distillation (bp. 220 °C). Ester **2** (1.61 g, 5.91 mmol, 97%) was obtained as a slightly yellow oil. TLC (petroleum ether/ethyl acetate, 9/1, *v/v*): R_f = 0.28; Spectroscopic data in agreement with the literature.[S6] ^{1}H-NMR (400 MHz, $CDCl_3$): δ (ppm) = 7.86 (d, *J*=8.7 Hz, 1H), 7.30 (t, *J*=7.9 Hz, 1H), 6.85 – 6.93 (m, 5H), 3.86 (s, 3H), 3.83 (s, 3H), 3.63 (s, 3H).

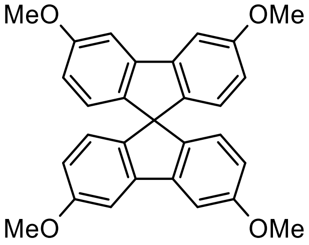

3,3',6,6'-Tetramethoxy-9,9'-spirobifluorene (5)

Spirobifluorene **5** was synthesized similarly to a literature-known procedure.[S6] For this, dibromide **1** (2.20 g, 5.91 mmol, 1.0 equiv) was dissolved in dry diethylether (55 mL) in a

Schlenk flask under N_2 atmosphere and cooled to -20 °C. Over 10 min, *n*-BuLi in hexane (2.27 M, 5.3 mL, 12.0 mmol, 2.0 equiv) was added, and the mixture stirred for another 50 min. Afterwards, biphenyl ester **2** was dissolved in dry diethylether (28 mL) and added dropwise to the reaction mixture at -20 °C. After completed addition, the mixture was stirred for another 30 min at -20 °C and then left to warm to room temperature within 2 h. Water (50 mL) was added, and the aqueous phase was extracted thrice with diethylether (30 mL). Combined organic extracts were washed with water (30 mL) and sat. NaCl solution (30 mL), dried over Na_2SO_4, filtered and the ether removed *in vacuo*. The residue was taken up in acetic acid (50 mL) and conc. HCl (5 mL) and refluxed for 2 h (140 °C oil bath temperature). Water (30 mL) was added, and the solution was extracted with ethyl acetate (3 × 10 mL). The combined organic extracts were washed with sat. $NaHCO_3$ (3 × 30 mL) and sat. NaCl (3 × 30 mL), dried over sodium sulfate, filtered, and the solvent removed under reduced pressure. After recrystallization from chloroform/*n*-hexane, tetraether **5** (581 mg, 1.33 mmol, 23%) was obtained as colorless prisms. TLC (petroleum ether/dichloromethane, 2/3, *v/v*): R_f = 0.33; Spectroscopic data in agreement with the literature.[S6] ^{1}H-NMR (400 MHz, $CDCl_3$): δ (ppm) = 7.30 (d, *J*=2.4 Hz, 4H), 6.66 (dd, *J*=2.4, 8.3 Hz, 4H), 6.62 (d, *J*=8.3 Hz, 4H), 3.88 (s, 24H).

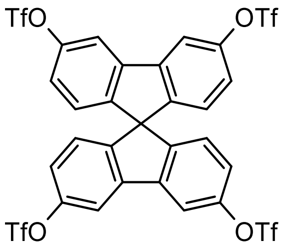

3,3',6,6'-Tetrakis[(trifluoromethyl)sulfonyloxy)-9,9'-spirobifluorene (6)

Tetramethylether **5** was demethylated and converted to the tetratriflate **6** essentially as described in the literature.[S6] Spirobifluorene **5** (768 mg, 1.76 mmol, 1.0 equiv) was dissolved in dry DCM (15 mL) under nitrogen and cooled to 0 °C. While stirring, BBr_3 (1M in DCM, 21.1 mL, 21.1 mmol, 12.0 equiv) was added dropwise over 30 min, and the mixture was kept at 0 °C for another 30 min. The solution was stirred for 20 h at room temperature and then cooled again to 0 °C. Dry methanol (24 mL) was slowly added, and all volatile components were removed under reduced pressure to yield 3,3',6,6'-tetrahydroxy-9,9'-spirobifluorene, which was used in the subsequent triflatization without further purification. An analytical sample gave TLC (petroleum ether/ethyl acetate, 2/3, *v/v*): R_f = 0.10. The demethylated spirobifluorene was dissolved in dry pyridine (16 mL) under nitrogen and cooled to -20 °C. Trifluoromethane-sulfonic anhydride (1.9 mL, 11.3 mmol, 6.4 equiv) was added slowly and the reaction mixture was stirred for 1 h at -20 °C and another 22 h at room temperature. Water (20 mL) was added, and the aqueous phase was extracted with diethyl ether (3 × 15 mL). The combined extracts were washed with water (15 mL) and brine (15 mL), dried over Na_2SO_4, filtered and the solvent removed under reduced pressure. The residue was purified via column chromatography (silica, petroleum ether/dichloromethane, 4/1 - 3/1, *v/v*) and recrystallized from chloroform/petroleum ether. The solid thus obtained was dissolved in chloroform and filtered, the solvent of the filtrate was removed *in vacuo* and the tetratriflate **6** (805 mg, 886 µmol, 50% over 2 steps) was obtained as a colorless solid. TLC (petroleum ether/dichloromethane, 4/1, *v/v*): R_f = 0.23. Spectroscopic data was in agreement with the literature.[S6] ^{1}H-NMR (400 MHz, $CDCl_3$): δ (ppm) = 7.75 (d, *J*=2.4 Hz, 4H), 7.13 (dd, *J*=2.4, 8.3 Hz, 4H), 6.83 (d, *J*=8.4 Hz, 4H).

Suzuki-Miyaura Cross-Coupling to Tetraarylspirobifluorenes (General Protocol I)

The following protocol is for the synthesis of 3,3',6,6'-tetrakis(2,4-dimethoxyphenyl)-9,9'-spirobifluorene (**7**), and is representative.

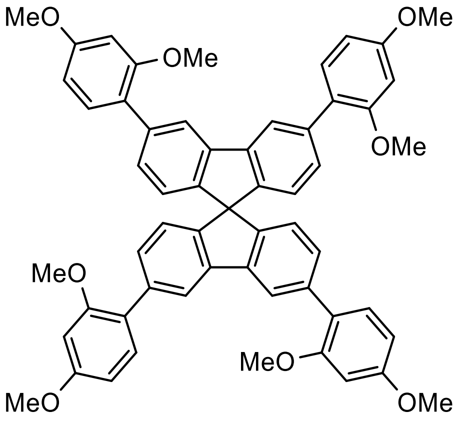

In a flame-dried Schlenk flask, tetratriflate **6** (200 mg, 220 µmol, 1.0 equiv), 2,4-dimethoxyphenylboronic acid (320 mg, 1.76 mmol, 8.0 equiv), potassium carbonate (396 mg, 2.87 mmol, 13 equiv), tricyclohexylphosphine (19 mg, 66 µmol, 0.3 equiv), and $Pd_2(dba)_3$ (10 mg, 11 µmol, 5 mol%) were added to a degassed mixture of toluene (1.8 mL), ethanol (0.2 mL), and water (0.7 mL), which was further degassed via three freeze-pump-thaw cycles. The mixture was stirred at 80 °C for 21 h, left to cool to room temperature and diluted with DCM (10 mL). The organic phase was washed with water (5 mL) and sat. NH_4Cl solution (5 mL), dried over Na_2SO_4, filtered and purified via column chromatography (silica, petroleum ether / DCM, 1/3, *v/v*). The resulting white solid was taken up in DCM (5 mL) and precipitated by adding methanol (5 mL). The precipitate was isolated, dried in vacuo, and recrystallized from toluene to yield the compound **7** (104 mg, 121 µmol, 55%) as colourless prisms. TLC (petroleum ether/dichloromethane, 3/1, *v/v*): R_f = 0.19; ^{1}H-NMR (400 MHz, $CDCl_3$): δ (ppm) = 8.03 (d, *J*=1.2 Hz, 4H), 7.30 – 7.36 (m, 4H), 7.31 (dd, *J*=1.7, 8.0 Hz, 4H), 6.84 (d, *J*=8.0 Hz, 4H), 6.60 – 6.68 (m, 8H), 3.85 (d, *J*=6.2 Hz, 24H); ^{13}C-NMR (101 MHz, $CDCl_3$): δ (ppm) = 160.9, 158.0, 142.8, 142.2, 138.6, 131.7, 129.6, 123.8, 123.6, 121.6, 105.2, 99.2, 65.6, 55.9, 55.8; HR-EIMS *m/z* calc. for $C_{57}H_{48}O_8$ $[M]^+$ 860.334, found 860.332.

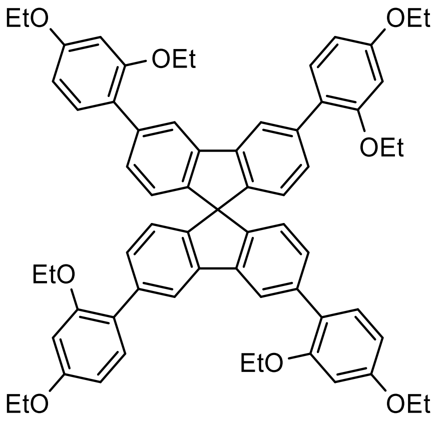

3,3',6,6'-Tetrakis(2,4-diethoxyphenyl)-9,9'-spirobifluorene (8)

Following General Protocol I, tetratriflate **6** (200 mg, 220 µmol, 1.0 equiv), 2,4-diethoxyphenylboronic acid (515 mg, 1.76 mmol, 8.0 equiv), potassium carbonate (243 mg, 1.76 mmol, 8.0 equiv), $Pd_2(dba)_3$ (8 mg, 9 µmol, 4 mol%), and XPhos (17 mg, 35 µmol, 16 mol%) were added to a degassed mixture of toluene (1.8 mL) and water (0.4 mL). The mixture was stirred at 80 °C for 4 h. Purified via column chromatography (silica, petroleum ether / DCM, 2/3 - 1/3, *v/v*). The resulting white solid was taken up in DCM (5 mL) and precipitated by adding methanol (5 mL). The precipitate was isolated, dried in vacuo, and recrystallized from chloroform/*n*-hexane to yield the product **8** (194 mg, 199 µmol, 91%) as colourless needles. TLC (petroleum ether/dichloromethane, 1/2, *v/v*): $R_f = 0.42$; ^{1}H-NMR (400 MHz, $CDCl_3$): δ (ppm) = 8.05 (d, J=1.3 Hz, 4H), 7.31 – 7.38 (m, 4H), 7.33 (dd, J=1.7, 7.9 Hz, 4H), 6.89 (d, J=7.9 Hz, 4H), 6.52 – 6.60 (m, 8H), 4.08 (quin, J=7.0 Hz, 16H), 1.46 (t, J=6.8 Hz, 12H), 1.40 (t, J=6.8 Hz, 12H); ^{13}C-NMR (101 MHz, $CDCl_3$): δ (ppm) = 159.6, 157.0, 147.6, 141.9, 137.9, 131.5, 129.1, 123.9, 123.8, 120.9, 105.5, 100.7, 65.4, 64.2, 63.7, 15.03, 14.95; HR-EIMS *m/z* calc. for $C_{65}H_{64}O_8$ $[M]^+$ 972.4596, found 972.4595.

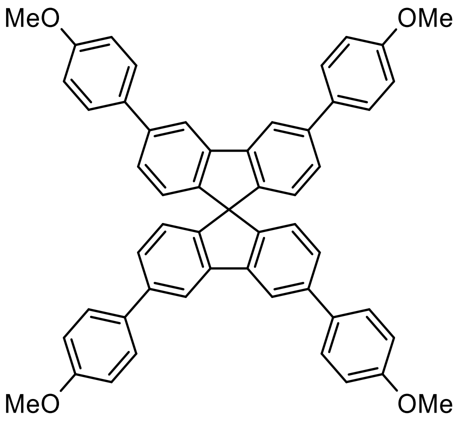

3,3',6,6'-Tetrakis(4-methoxyphenyl)-9,9'-spirobifluorene (9)

Following General Protocol I, tetratriflate **6** (128 mg, 141 µmol, 1.0 equiv), 4-methoxyphenylboronic acid (171 mg, 1.13 mmol, 8.0 equiv), potassium carbonate (253 mg, 1.83 mmol, 13.0 equiv), $Pd_2(dba)_3$ (6 mg, 7 µmol, 5 mol%), and tricyclohexylphosphine (12 mg, 42 µmol, 0.3 equiv) were added to a degassed mixture of toluene (1.1 mL), EtOH (0.1 mL), and water (0.5 mL). The mixture was stirred at 80 °C for 3 h. Purified via column chromatography (silica, petroleum ether / DCM, 2/1 - 1/1, *v/v*) to yield the product **9** (98 mg, 132 µmol, 94%) as colourless solid. TLC (petroleum ether/dichloromethane, 2/1, *v/v*): R_f = 0.15; ^{1}H-NMR (400 MHz, $CDCl_3$): δ (ppm) = 8.10 (d, *J*=1.2 Hz, 4H), 7.63 (d, *J*=8.7 Hz, 8H), 7.34 (dd, *J*=1.5, 7.9 Hz, 4H), 7.02 (d, *J*=8.7 Hz, 8H), 6.85 (d, *J*=7.9 Hz, 4H), 3.87 (s, 12H); ^{13}C-NMR (101 MHz, $CDCl_3$): δ (ppm) = 159.3, 147.8, 142.4, 140.8, 134.0, 128.4, 126.9, 124.5, 118.4, 114.4, 65.1, 55.5; HR-EIMS *m/z* calc. for $C_{53}H_{40}O_4$ $[M]^+$ 740.2921, found 740.2925.

Rothemund Reaction (General Protocol II)

The following protocol is for the synthesis of 5,10,15,20-tetrakis(2-bromo-4-methoxyphenyl)porphyrin (**14)**, and is representative.

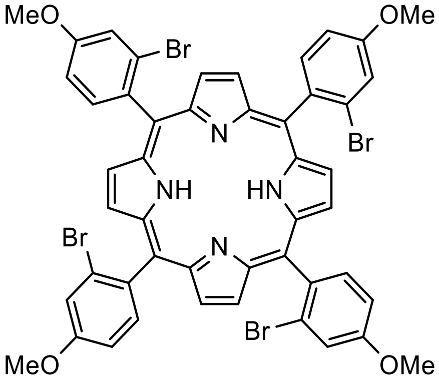

The synthesis is a slight modification of a published protocol.[S7] In a two-neck round-bottom flask, propionic acid (18 mL) was stirred at 150 °C. Freshly distilled pyrrole (347 µL, 355 mg, 5.00 mmol, 1.0 equiv) and 2-bromo-4-methoxybenzaldehyde (1.08 g, 5.0 mmol, 1.0 equiv) were dissolved in propionic acid (2 mL) and added dropwise to the refluxing solution. The black suspension was stirred at 150 °C for 1 h, allowed to reach room temperature, diluted with water (100 mL) and extracted with $CHCl_3$ (3 × 50 mL). The combined organic phases were washed with sat. $NaHCO_3$ and sat. NaCl solution (50 mL each), dried over Na_2SO_4 and filtered. Silica (2 g) was added, and the black suspension was mixed at 60 °C for 1 h. The solvent was removed under reduced pressure, and the black residue purified via column chromatography (silica, $CHCl_3$/ethyl acetate 100/10 to 0/100, *v/v*). The resulting purple solid was recrystallized from MeOH (20 mL) to obtain target compound **14** (187 mg, 178 µmol, 14%) as dark shiny crystals. TLC (petroleum ether/dichloromethane, 1/1, *v/v*): $R_f = 0.29$, ^{1}H-NMR (400 MHz, $CDCl_3$): δ (ppm) = 8.70 (s, 8H, β-H), 8.16 – 7.93 (m, 4H, *ortho*-H_{ar}), 7.58 – 7.54 (m, 4H, *meta*-H_{ar}), 7.25 – 7.18 (m, 4H, *meta*-H_{ar}), 4.09 (s, 3H, OCH_3), -2.66 (br s, 2H, N*H*). ^{13}C-NMR (101 MHz, $CDCl_3$): δ (ppm) = 160.4, 160.4, 135.6, 134.9, 127.9, 127.6, 118.4, 117.1, 112.2, 56.0. HR-ESIMS *m/z* calc. for $C_{48}H_{37}Br_4N_4O_4$ $[M+H]^+$ 1050.9353, found 1050.9380.

Metallation of porphyrins (General Protocol III)

The following protocol is for the synthesis of [5,10,15,20-tetraphenylporphyrinato]zinc(II) (**24)**, and is representative.

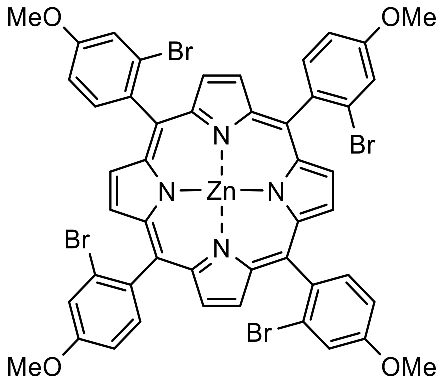

The synthesis is a modification of a published protocol.[S8] A round-bottom flask was charged with 5,10,15,20-tetrakis(2-bromo-4-methoxyphenyl)porphyrin **14** (50.0 mg, 47.6 µmol, 1.0 equiv) and zinc acetate (10.9 mg, 59.5 µmol, 1.3 equiv). Then, $CHCl_3$ (5 mL) was added, and the resulting mixture was stirred at 70 °C for 4 h. The pink solution was allowed to reach room temperature and directly purified via column chromatography (silica, $CHCl_3$/ethyl acetate 100/0 to 90/10, *v/v*) to obtain compound **24** (53 mg, quant.) as pink prisms. TLC ($CHCl_3$): R_f = 0.67. ^{1}H-NMR (400 MHz, $CDCl_3$): δ (ppm) = 8.70 (s, 8H, β-H), 8.21 – 7.93 (m, 4H, *ortho-H*$_{ar}$), 7.60 – 7.49 (m, 4H, *meta-H*$_{ar}$), 7.25 – 7.15 (m, 4H, *meta-H*$_{ar}$), 4.09 (s, 3H, OCH_3). ^{13}C-NMR (101 MHz, $CDCl_3$): δ (ppm) = 160.2, 160.2, 150.5 135.6, 135.5, 131.8, 119.3, 117.0, 112.1, 56.0. HR-ESIMS: *m/z* calc. for $C_{48}H_{33}Br_4ZnN_4O_4$ $[M+H]^+$ 1114.8467, found 1114.8455.

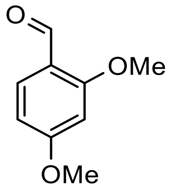

2,4-Dimethoxybenzaldehyde

The synthesis followed a published protocol.[S9] A round-bottom flask with 2,4-dihydroxybenzaldehyde (5.00 g, 36.2 mmol, 1.0 equiv) and potassium carbonate (20.1 g, 145 mmol, 4.0 equiv) was treated with acetone (100 mL), and the colorless suspension was stirred at 50°C for 30 min. Iodomethane (4.96 mL, 11.3 g, 79.6 mmol, 2.2 equiv) was added, and the yellow suspension was stirred at 65 °C for 24 h. The mixture was allowed to reach

room temperature, filtered and washed with ethyl acetate (250 mL). After removing the solvents under reduced pressure, the residue was taken up in ethyl acetate (100 mL) and washed with water, 2 M NaOH and sat. NaCl solution (100 mL each). The organic phase was dried over Na_2SO_4, filtered and the solvent was removed under reduced pressure. The yellow residue was purified via column chromatography (silica, petroleum ether/ethyl acetate 1/1, *v/v*) to yield the diether (4.94 g, 29.7 mmol, 82%) as colorless needles. TLC (petroleum ether/dichloromethane, 1/1, *v/v*): R_f = 0.11. Spectroscopic data were in agreement with the literature.[S10] ^{1}H-NMR (400 MHz, $CDCl_3$): δ (ppm) = 10.3 (s, 1H), 7.81 (d, J = 8.6 Hz, 1H), 6.55 (dd, J = 8.7, 2.3 Hz, 1H), 6.45 (d, J = 2.2 Hz, 1H), 3.90 (s, 3H), 3.88 (s, 3H).

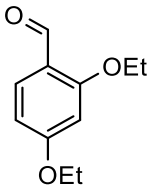

2,4-Diethoxybenzaldehyde

The synthesis followed a published protocol.[S9] A round-bottom flask was charged with 2,4-dihydroxybenzaldehyde (10.0 g, 72.4 mmol, 1.0 equiv), potassium carbonate (40.0 g, 290 mmol, 4.0 equiv) and potassium iodide (2.40 g, 14.5 mmol, 20 mol%). Acetone (250 mL) was added and the colorless suspension was stirred at 50°C for 30 min. Bromoethane (11.9 mL, 17.4 g, 159 mmol, 2.2 equiv) was added and the yellow suspension was stirred at 65 °C for 24 h. The mixture was allowed to cool to room temperature, filtered and washed with ethyl acetate (500 mL). After removing the solvents under reduced pressure, the residue was taken up in ethyl acetate (200 mL) and washed with water, 2 M NaOH and sat. NaCl solution (200 mL each). The organic phase was dried over Na_2SO_4, filtered and the solvent was removed under reduced pressure. The yellow residue was purified via column chromatography (silica, petroleum ether / ethyl acetate 1/1, *v/v*) to yield the diether (12.2 g, 62.6 mmol, 86%) as colorless needles. TLC (petroleum ether/dichloromethane, 1/1, *v/v*): R_f = 0.27. Spectroscopic data in agreement with the literature.[S9] ^{1}H-NMR (400 MHz, $CDCl_3$): δ (ppm) = 10.3 (s, 1H), 7.80 (d, J = 8.7 Hz, 1H), 6.52 (dd, J = 8.7, 2.2 Hz, 1H), 6.42 (d, J = 2.2 Hz, 1H), 4.15 – 4.06 (m, 4H), 1.47 (t, J = 7.0 Hz, 3H), 1.44 (t, J = 7.0 Hz, 3H).

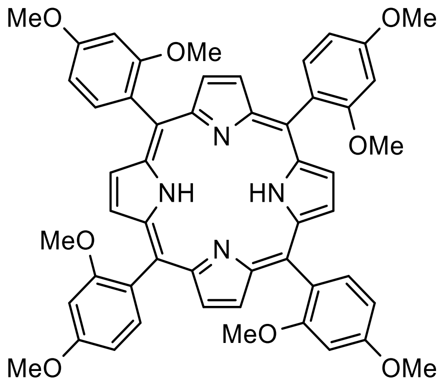

5,10,15,20-Tetrakis(2,4-dimethoxyphenyl)porphyrin (10)

The synthesis of the title compound followed General Protocol II, using 2,4-dimethoxybenzaldehyde (831 mg, 5.00 mmol, 1.0 equiv), pyrrole (347 µL, 336 mg, 5.00 mmol, 1.0 equiv) and propionic acid (20 mL). Compound **10** (195 mg, 228 µmol, 18%) was obtained as dark shiny crystals. TLC (petroleum ether/dichloromethane, 1/1, *v/v*): R_f = 0.32. Spectroscopic data was in agreement with the literature.[S11] ^{1}H-NMR (400 MHz, $CDCl_3$): δ (ppm) = 8.74 (s, 8H), 7.95 – 7.79 (m, 4H), 6.90 – 6.84 (m, 8H), 4.09 (s, 12H), 3.60 – 3.52 (m, 12H), -2.63 (br s, 2H).

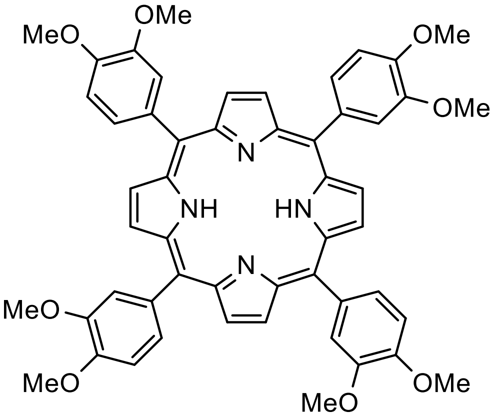

5,10,15,20-Tetrakis(3,4-dimethoxyphenyl)porphyrin (11)

To synthesize the title compound, following the General Protocol II, 3,4-dimethoxybenzaldehyde (1.66 mg, 10.0 mmol, 1.0 equiv), pyrrole (694 mL, 671 g, 10.0 mmol, 1.0 equiv) und propionic acid (40 mL) were used. Compound **11** (251 mg, 228 µmol, 12%) was obtained as dark shiny crystals. TLC (chloroform/ethyl acetate, 99/1, *v/v*): R_f = 0.11. Spectroscopic data in agreement with the literature.[S12] ^{1}H-NMR (500 MHz, $CDCl_3$): *δ* (ppm) = 8.91 (s, 8H), 7.81 – 7.74 (m, 8H), 7.28 – 7.23 (d, *J* = 8.2 Hz, 4H), 4.18 (s, 12H), 3.99 (s, 12H), -2.73 (s, 2H).

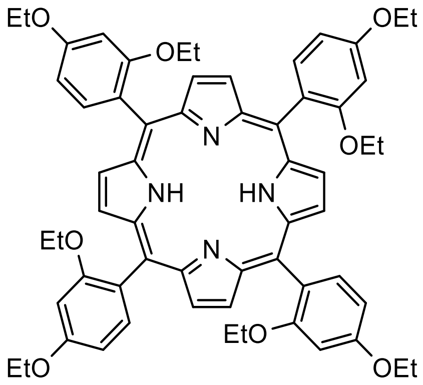

5,10,15,20-Tetrakis(2,4-diethoxyphenyl)porphyrin (12)

Following the General Protocol II, 2,4-diethoxybenzaldehyde (417 mg, 2.15 mmol, 1.0 equiv), pyrrole (150 µL, 145 mg, 2.15 mmol, 1.0 equiv) und propionic acid (20 mL) used to synthesize target molecule. Compound **12** (57 mg, 59.8 µmol, 11%) was obtained as dark shiny crystals. TLC (petroleum ether/dichloromethane, 1/1, *v/v*): R_f = 0.39. ^{1}H-NMR (400 MHz, $CDCl_3$): δ (ppm) = 8.77 (s, 8H, β-*H*), 7.92 – 7.82 (m, 4H, *ortho*-H_{ar}), 6.91 – 6.82 (m, 8H, *meta*-H_{ar}), 4.32 (q, *J* = 6.9 Hz, 8H, *para*-OCH_2CH_3), 3.90 (q, *J* = 7.0 Hz, 8H, *ortho*-OCH_2CH_3), 1.61 (t, *J* = 6.9 Hz, 12H, *para*-OCH_2CH_3), 0.71 – 0.62 (m, 12H, *ortho*-OCH_2CH_3), -2.65 (br s, 2H, N*H*). ^{13}C-NMR (101 MHz, $CDCl_3$): δ (ppm) = 159.8, 159.7, 136.3, 124.6, 124.6, 115.6, 115.6, 104.3, 100.3, 64.4, 64.4, 14.5, 14.5. HR-ESIMS: *m/z* calc. for $C_{60}H_{63}N_4O_8$ [M+H]$^+$: 967.4640, found 967.4631.

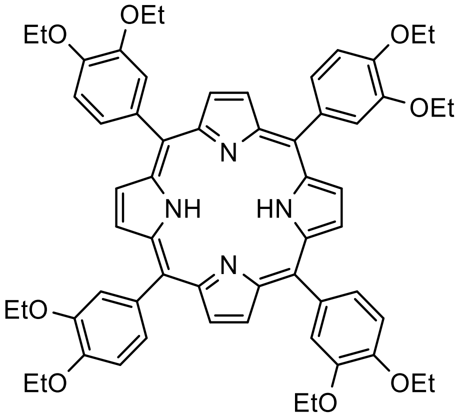

5,10,15,20-Tetrakis(3,4-diethoxyphenyl)porphyrin (13)

Target compound was synthesized following the General Protocol II, using 3,4-diethoxybenzaldehyde (1.77 mg, 1.94 g, 10.0 mmol, 1.0 equiv), pyrrole (694 mL, 671 g, 10.0 mmol, 1.0 equiv) und propionic acid (40 mL). Compound **13** (251 mg, 272 µmol, 11%) was obtained as dark shiny crystals. TLC (chloroform/ethyl acetate, 99/1, *v*/*v*): R_f = 0.18. Spectroscopic data in agreement with the literature.[S13] ^{1}H-NMR (400 MHz, $CDCl_3$): δ (ppm) = 8.90 (s, 8H), 7.78 (s, 4H), 7.72 (d, *J* = 8.3 Hz, 4H), 7.26 – 7.23 (m, 4H) 4.40 (q, *J* = 7.0 Hz, 8H), 4.23 (q, *J* = 7.0 Hz, 8H), 1.67 (t, *J* = 7.0 Hz, 12H), 1.55 – 1.46 (m, 12H), -2.74 (br s, 2H).

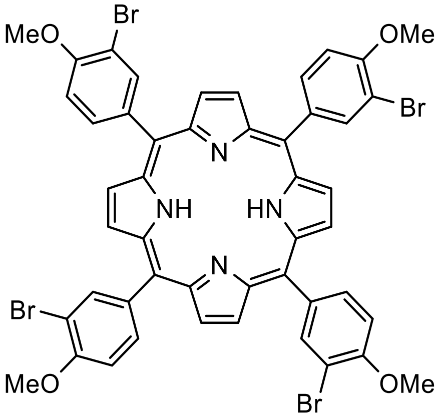

5,10,15,20-Tetrakis(3-bromo-4-methoxyphenyl)porphyrin (15)

Target molecules was synthesized following the General Protocol II, using 3-bromo-4-methoxybenzaldehyde (3.23 mg, 15.0 mmol, 1.0 equiv), pyrrole (1.04 mL, 1.01 g, 15.0 mmol, 1.0 equiv) und propionic acid (60 mL). The target compound **15** (327 mg, 311 µmol, 8%) was obtained as dark shiny crystals. TLC (chloroform): R_f = 0.84. ^{1}H-NMR (400 MHz, $CDCl_3$): δ (ppm) = 8.88 (s, 8H, β-*H*), 8.42 (d, *J* = 2.2 Hz, 4H, *H*-2), 8.10 (dd, *J* = 8.3, 2.2 Hz, 4H, *H*-6), 7.26 (d, *J* = 8.9 Hz, 4H, *H*-5), 4.20 (s, 12H, OC*H*3), -2.85 (br s, 2H, N*H*). ^{13}C-NMR (101 MHz, $CDCl_3$): δ (ppm) = 155.9, 155.9, 138.9, 138.8, 135.7, 135.6, 134.6, 118.6, 110.1, 56.7. HR-EIMS: *m/z* calc. for $C_{48}H_{36}Br_4N_4O_4$ [$M^{+\cdot}$] 1050.9353, found 1050.9348.

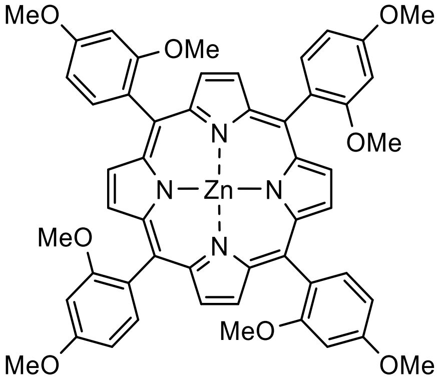

[5,10,15,20-Tetrakis(2,4-dimethoxyphenyl)porphyrinato]zinc(II) (16)

The synthesis of the title compound followed General Protocol III, using 5,10,15,20-tetrakis(2,4-dimethoxyphenyl)porphyrin **10** (10.0 mg, 11.7 µmol, 1.0 equiv), zinc(II) acetate (2.68 mg, 14.6 µmol, 1.3 equiv). Stirred in chloroform (1 mL) at 70 °C for 7 h. After purification via column chromatography (silica, chloroform/ethyl acetate 100/0 to 90/10, *v/v*), compound **16** (11 mg, quant.) was obtained as violet prisms. TLC (chloroform): $R_f = 0.14$. ^{1}H-NMR (400 MHz, $CDCl_3$): δ (ppm) = 8.75 (s, 8H, β-*H*), 7.98 – 7.73 (m, 4H, *ortho*-H_{ar}), 6.97 – 6.73 (m, 8H, *meta*-H_{ar}), 4.09 (s, 12H, *para*-OCH_3), 3.67 – 3.49 (m, 12H, *ortho*-OCH_3). ^{13}C-NMR (101 MHz, $CDCl_3$): δ (ppm) = 161.1, 160.4, 150.7, 136.1, 131.5, 124.8, 116.2, 103.4, 98.5, 56.0, 55.8. HR-EIMS: *m/z* calc. for $C_{52}H_{44}N_4O_8$ [$M^{+\cdot}$] 916.2445, found 916.2454.

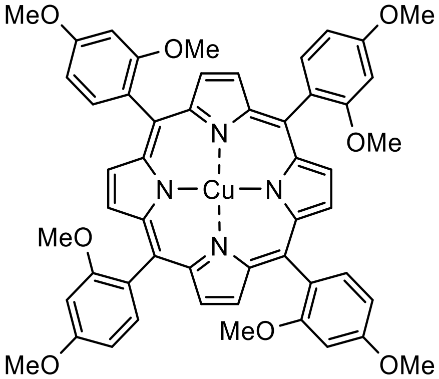

[5,10,15,20-Tetrakis(2,4-dimethoxyphenyl)porphyrinato]copper(II) (17)

Following the General Protocol III, target molecule was synthesized using 5,10,15,20-tetrakis(2,4-dimethoxyphenyl)porphyrin **10** (10.0 mg, 11.7 µmol, 1.0 equiv), copper(II) acetate (2.66 mg, 14.6 µmol, 1.3 equiv) and chloroform (1 mL). Stirred at 70 °C for 5 h. After purification via column chromatography (silica, chloroform/ethyl acetate 100/0 to 90/10, *v/v*), compound **17** (6 mg, 6.5 µmol, 56%) was obtained as reddish prisms. TLC (chloroform): $R_f =$ 0.48. HR-EIMS: *m/z* calc. for $C_{52}H_{44}CuN_4O_8$ [$M^{+\cdot}$] 915.2450, found 915.2454.

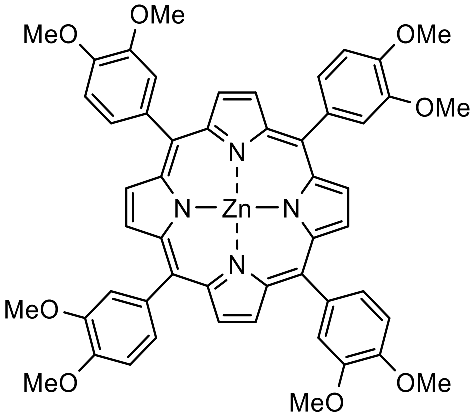

[5,10,15,20-Tetrakis(3,4-dimethoxyphenyl)porphyrinato]zinc(II) (18)

Following the General Protocol III, target molecule was synthesized by using 5,10,15,20-tetrakis(3,4-dimethoxyphenyl)porphyrin **11** (20.0 mg, 23.4 µmol, 1.0 equiv), zinc(II) acetate (5.36 mg, 29.2 µmol, 1.3 equiv) and chloroform (2 mL) and stirring at 70 °C for 10 h. After purification via column chromatography (silica, chloroform), compound **18** (21 mg, quant.) was obtained as violet prisms. TLC (chloroform/ethyl acetate, 99/1, *v/v*): R_f = 0.08. Spectroscopic data was in agreement with the literature.[S14] ^{1}H-NMR (400 MHz, $CDCl_3$): δ (ppm) = 9.03 (s, 8H), 7.82 – 7.72 (m, 8H), 7.25 – 7.20 (d, J = 8.1 Hz, 4H), 4.16 (s, 12H), 3.97 (s, 12H). HR-EIMS: *m/z* calc. for $C_{48}H_{32}Br_4CuN_4O_4$ [$M^{+\cdot}$] 1110.8414, found 1110.8429

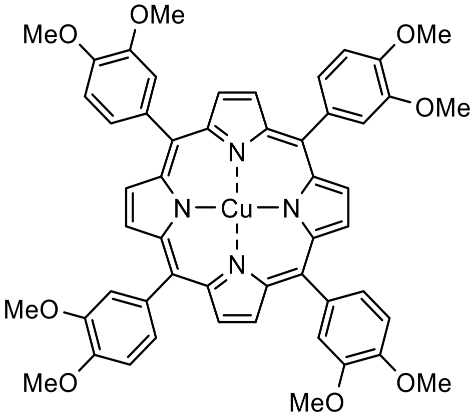

[5,10,15,20-Tetrakis(3,4-dimethoxyphenyl)porphyrinato]copper(II) (19)

Following the General Protocol III, 5,10,15,20-tetrakis(3,4-dimethoxyphenyl)porphyrin **11** (20.0 mg, 23.4 µmol, 1.0 equiv), copper(II) acetate (4.69 mg, 29.2 µmol, 1.3 equiv) and chloroform (2 mL) were stirred at 70 °C for 7 h. After purification via column chromatography (silica, chloroform/ethyl acetate, 98/2 to 96/4, *v/v*), compound **19** (21 mg, quant.) was obtained as violet prisms. TLC (chloroform/ethyl acetate, 99/1, *v/v*): R_f = 0.34. HR-EIMS: *m/z* calc. for $C_{52}H_{44}CuN_4O_8$ [$M^{+\cdot}$] 915.2455, found 915.2456.

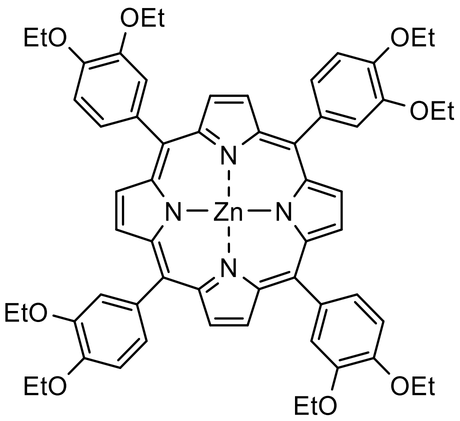

[5,10,15,20-Tetrakis(3,4-diethoxyphenyl)porphyrinato]zinc(II) (20)

Target compound was synthesized following the General Protocol III. 5,10,15,20-tetrakis(3,4-diethoxyphenyl)porphyrin **13** (20.0 mg, 20.7 µmol, 1.0 equiv), zinc(II) acetate (4.74 mg, 25.9 µmol, 1.3 equiv) and chloroform (2 mL) were stirred at 70 °C for 7 h. After purification via column chromatography (silica, chloroform/ethyl acetate, 100/0 to 98/2, *v/v*), compound **20** (20 mg, 19.4 µmol 94%) was obtained as violet prisms. TLC (chloroform/ethyl acetate, 99/1, *v/v*): R_f = 0.11. HR-EIMS: *m/z* calc. for $C_{48}H_{32}Br_4CuN_4O_4$ [$M^{+\cdot}$] 1110.8414, found 1110.8429. Spectroscopic data in agreement with the literature.[S13] ^{1}H-NMR (400 MHz, $CDCl_3$): δ (ppm) = 9.01 (s, 8H), 7.77 (br s, 4H), 7.70 (d, *J* = 8.0 Hz, 4H), 7.20 (d, *J* = 8.1 Hz, 4H), 4.37 (q, *J* = 6.9 Hz, 8H), 4.20 (q, *J* = 6.9 Hz, 8H), 1.66 (t, *J* = 7.0 Hz, 12H), 1.49 (t, *J* = 7.0 Hz, 12H).

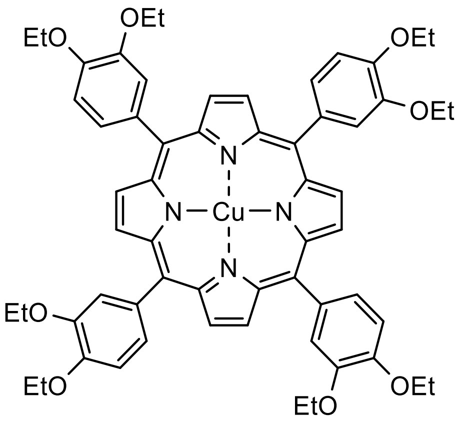

[5,10,15,20-Tetrakis(3,4-diethoxyphenyl)porphyrinato]copper(II) (21)

Following the General Protocol III, 5,10,15,20-tetrakis(3,4-diethoxyphenyl)porphyrin **13** (20.0 mg, 20.7 µmol, 1.0 equiv), copper(II) acetate (4.69 mg, 25.9 µmol, 1.3 equiv) and chloroform (2 mL) were stirred at 70 °C for 7 h. After purification via column chromatography (silica, chloroform/ethyl acetate, 100/0 to 98/2, *v/v*), compound **21** (21 mg, quant.) was obtained as reddish prisms. TLC (chloroform/ethyl acetate, 99/1, *v/v*): R_f = 0.36. HR-ESIMS: *m/z* calc. for $C_{60}H_{61}CuN_4O_8$ $[M+H]^+$ 1028.3780, found 1028.3782

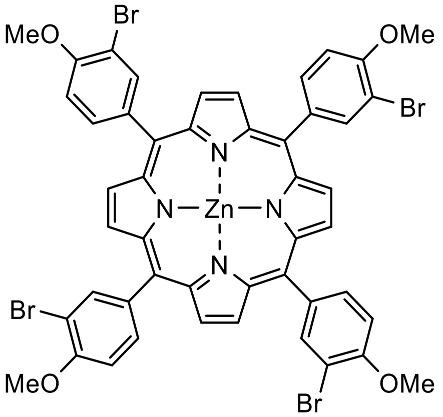

[5,10,15,20-Tetrakis(3-bromo-4-methoxyphenyl)porphyrinato]zinc(II) (22)

Following the General Protocol III, 5,10,15,20-tetrakis(3-bromo-4-methoxyphenyl)porphyrin **15** (20.0 mg, 19.0 µmol, 1.0 equiv), zinc(II) acetate (4.37 mg, 23.8 µmol, 1.3 equiv) and chloroform (2 mL) were stirred at 70 °C for 4 h. After purification via column chromatography (silica, chloroform), compound **22** (18 mg, 16.2 µmol, 85%) was obtained as violet prisms. TLC (chloroform): R_f = 0.84. ^{1}H-NMR (400 MHz, $CDCl_3$): δ (ppm) = 8.98 (s, 8H, β-*H*), 8.41 (br s, 4H, *H*-2), 8.09 (br s, 4H, *H*-6), 7.26 – 7.19 (m, 4H, *H*-5), 4.17 (s, 12H, OCH_3). ^{13}C-NMR (101 MHz, $CDCl_3$): δ (ppm) = 154.5, 149.4, 137.7, 135.3, 133.3, 131.0, 118.5, 109.1, 55.6.. HR-EIMS: *m/z* calc. for $C_{48}H_{32}Br_4ZnN_4O_4$ $[M^{+\cdot}]$ 1113.8389, found 1113.8388.

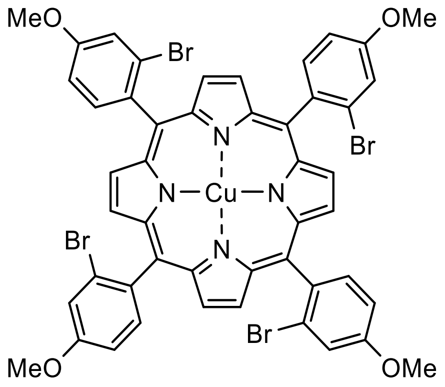

[5,10,15,20-Tetrakis(2-bromo-4-methoxyphenyl)porphyrinato]copper(II) (23)

Following the General Protocol III, 5,10,15,20-tetrakis(2-bromo-4-methoxyphenyl)porphyrin **14** (20.0 mg, 19.0 µmol, 1.0 equiv), copper(II) acetate (4.32 mg, 23.8 µmol, 1.3 equiv) and chloroform (2 mL) were stirred at 70 °C for 2 h. After purification via column chromatography (silica, chloroform/ethyl acetate 100/0 to 90/10, *v/v*), compound **23** (21 mg, quant.) was obtained as redish prisms. TLC (petroleum ether/chloroform, 1/1, *v/v*): $R_f = 0.31$. HR-ESIMS: *m/z* calc. for $C_{48}H_{33}Br_4CuN_4O_4$ $[M+H]^+$ 1111.8492, found 1111.8494.

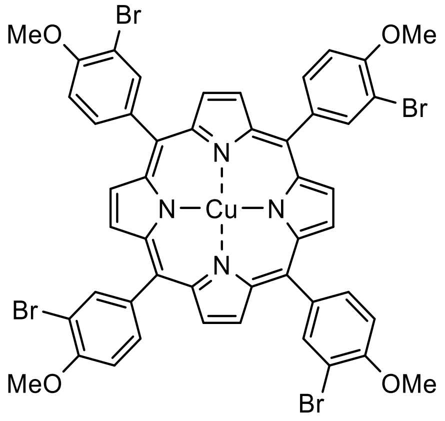

[5,10,15,20-Tetrakis(3-bromo-4-methoxyphenyl)porphyrinato]copper(II) (25)

Following the General Protocol III, 5,10,15,20-tetrakis(3-bromo-4-methoxyphenyl)porphyrin **15** (20.0 mg, 19.0 µmol, 1.0 equiv), copper(II) acetate (4.32 mg, 23.8 µmol, 1.3 equiv) and chloroform (2 mL) were stirred at 70 °C for 9 h. After purification via column chromatography (silica, chloroform), compound **25** (21 mg, quant.) was obtained as reddish prisms. TLC (chloroform): $R_f = 0.94$. HR-EIMS: *m/z* calc. for $C_{48}H_{32}Br_4CuN_4O_4$ $[M^{+\cdot}]$ 1110.8414, found 1110.8429.

Overview of yields of tetraarylporphyrins obtained by Rothemund reaction.

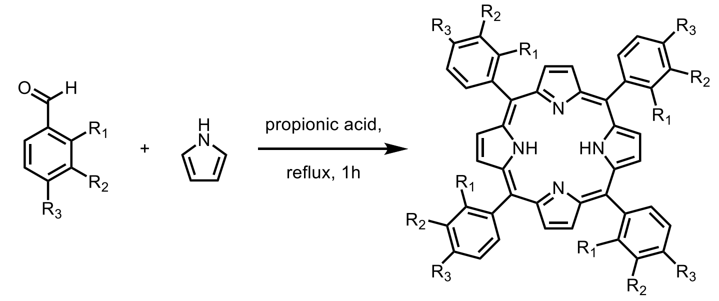

Structures of dipyrrylmethane fragments with the aryl substituents for each compound, shown here for clarity.

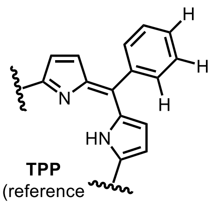
TPP
(reference
compound)

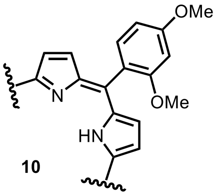
10

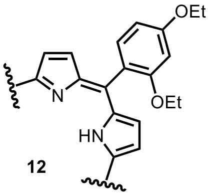
12

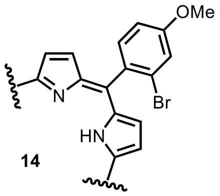
14

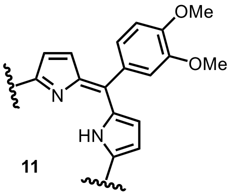
11

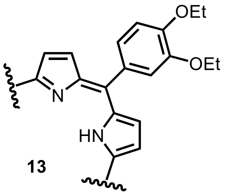
13

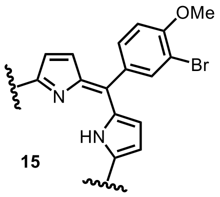
15

Table S1. Yields of the Rothemund reaction. Tetraphenylporphyrin is given as a reference.

Entry no.	Compound[a]	R_1	R_2	R_3	Yield [%]
1	**10**	OMe	H	OMe	18
2	**11**	H	OMe	OMe	12
3	**12**	OEt	H	OEt	11
4	**13**	H	OEt	OEt	11
5	**14**	Br	H	OMe	14
6	**15**	H	Br	OMe	8
7	**TPP**	H	H	H	23

[a] Prepared using the protocol given above.

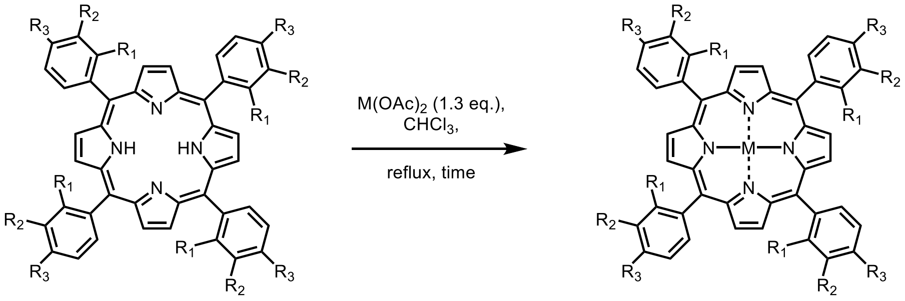

Table S2. Yields of tetraarylporphyrin metallates, as obtained from free bases and acetate salts.

Entry no.	Starting material	Reaction time [h]	M^{2+}	Yield [%]	Product[a]
1	**10**	7	Zn	quant.	**16**
2	**10**	5	Cu	56	**17**
3	**11**	10	Zn	quant.	**18**
4	**11**	10	Cu	84	**19**
5	**13**	7	Zn	94	**20**
6	**13**	7	Cu	quant.	**21**
7	**14**	4	Zn	quant.	**22**
8	**14**	2	Cu	quant.	**23**
9	**15**	4	Zn	85	**24**
10	**15**	9	Cu	quant.	**25**
11	**TPP**	18	Zn	quant.	**Zn-TPP**
12	**TPP**	2	Cu	quant.	**Cu-TPP**
13	**TPP**	24	Pd	40	**Pd-TPP**

[a] Prepared as described in the metallation protocol given above.

3. NMR Spectra

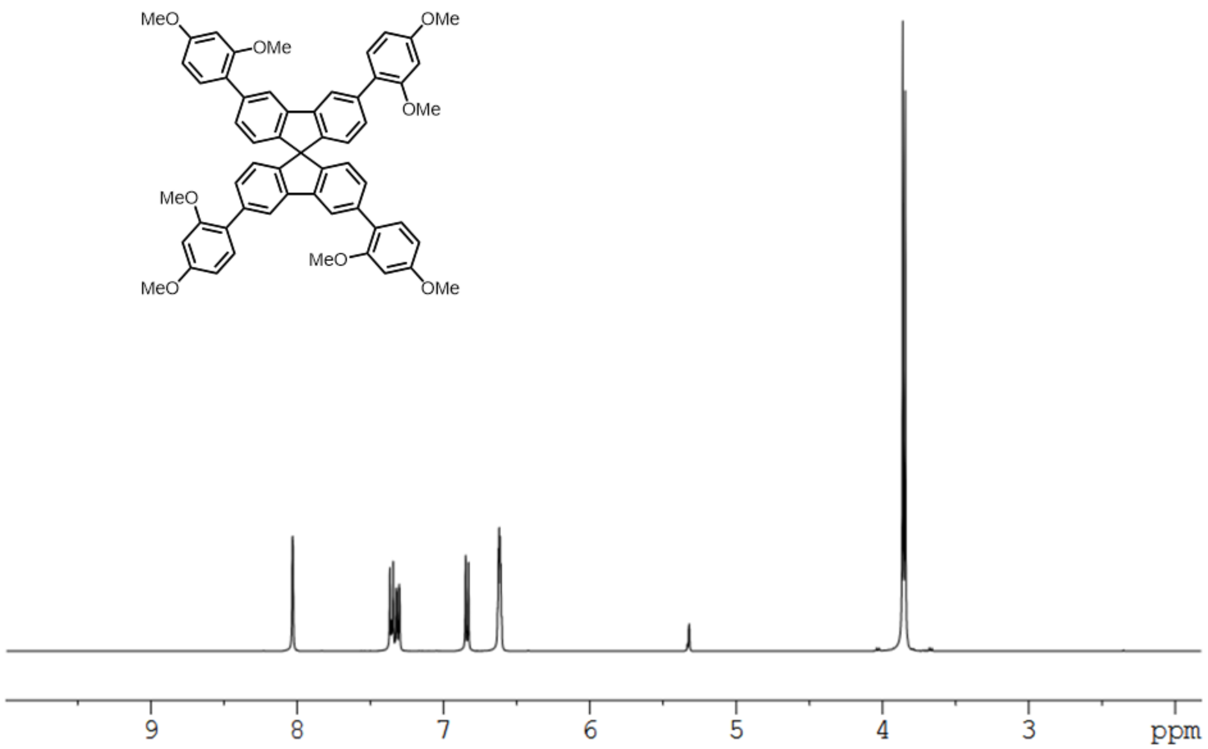

Figure S1. ^{1}H-NMR spectrum of compound **7** (CD_2Cl_2, 400 MHz).

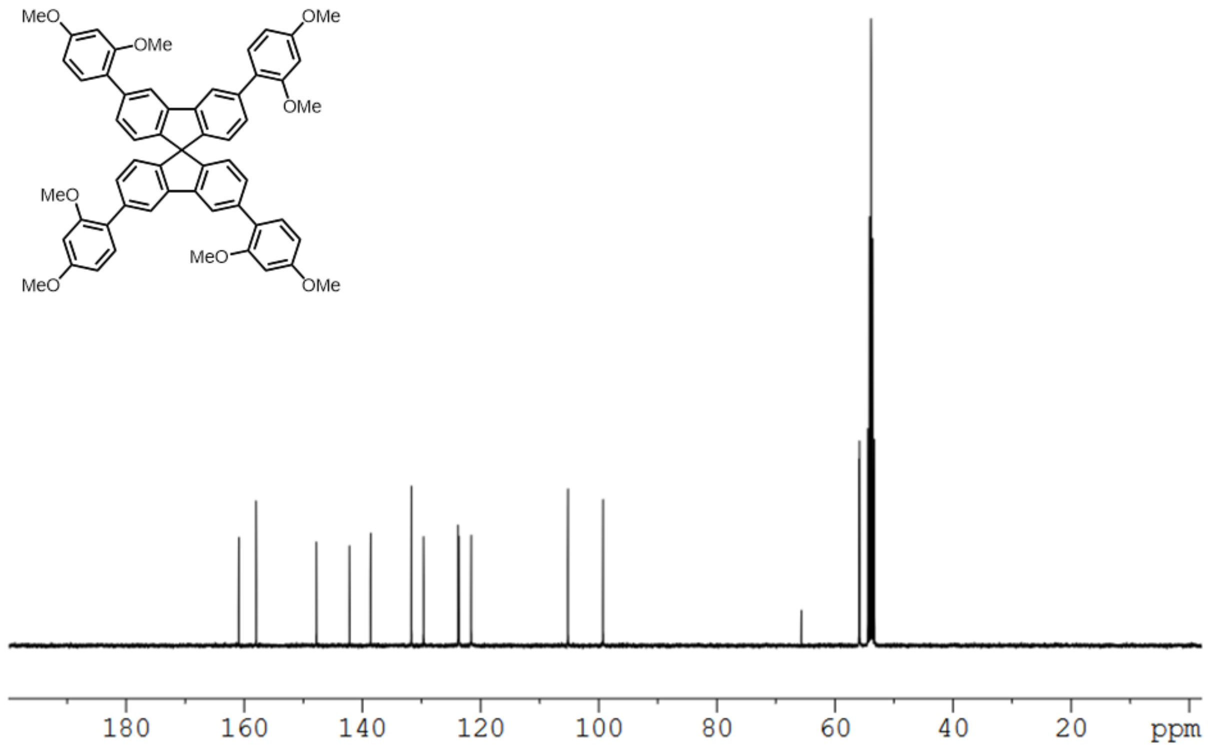

Figure S2. ^{13}C-NMR spectrum of compound **7** (CD_2Cl_2, 101 MHz).

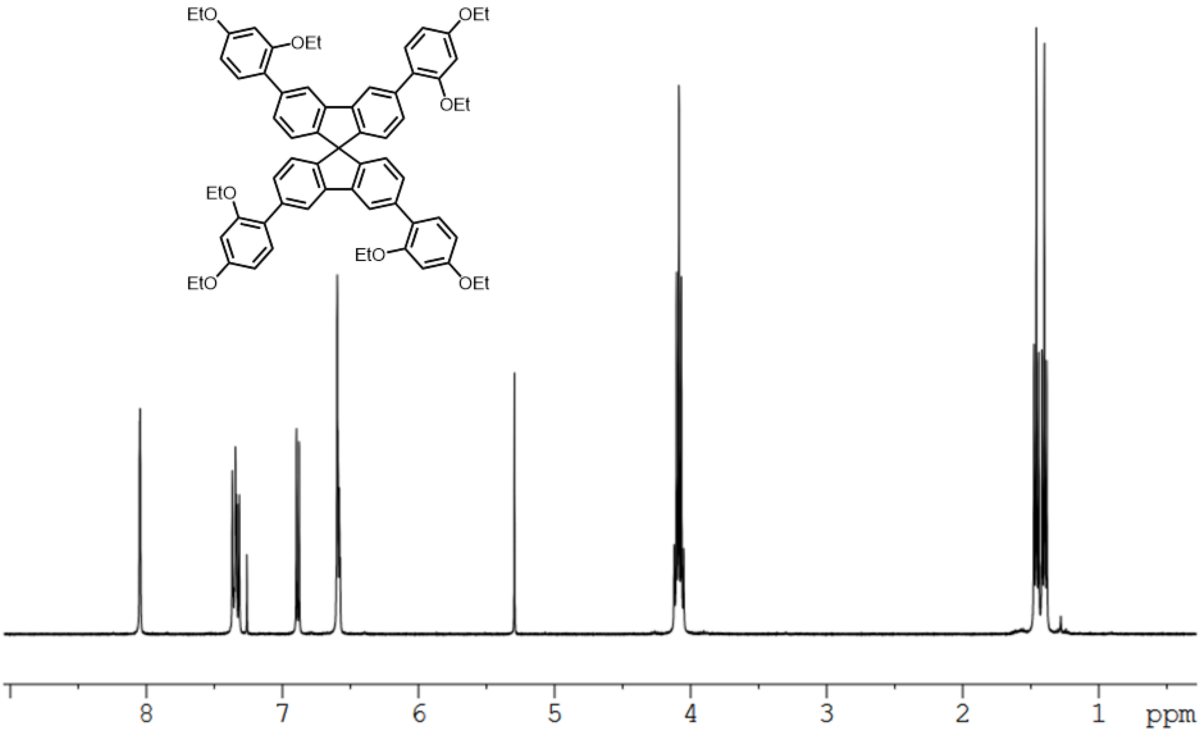

Figure S3. ^{1}H-NMR spectrum of compound **8** ($CDCl_3$, 400 MHz).

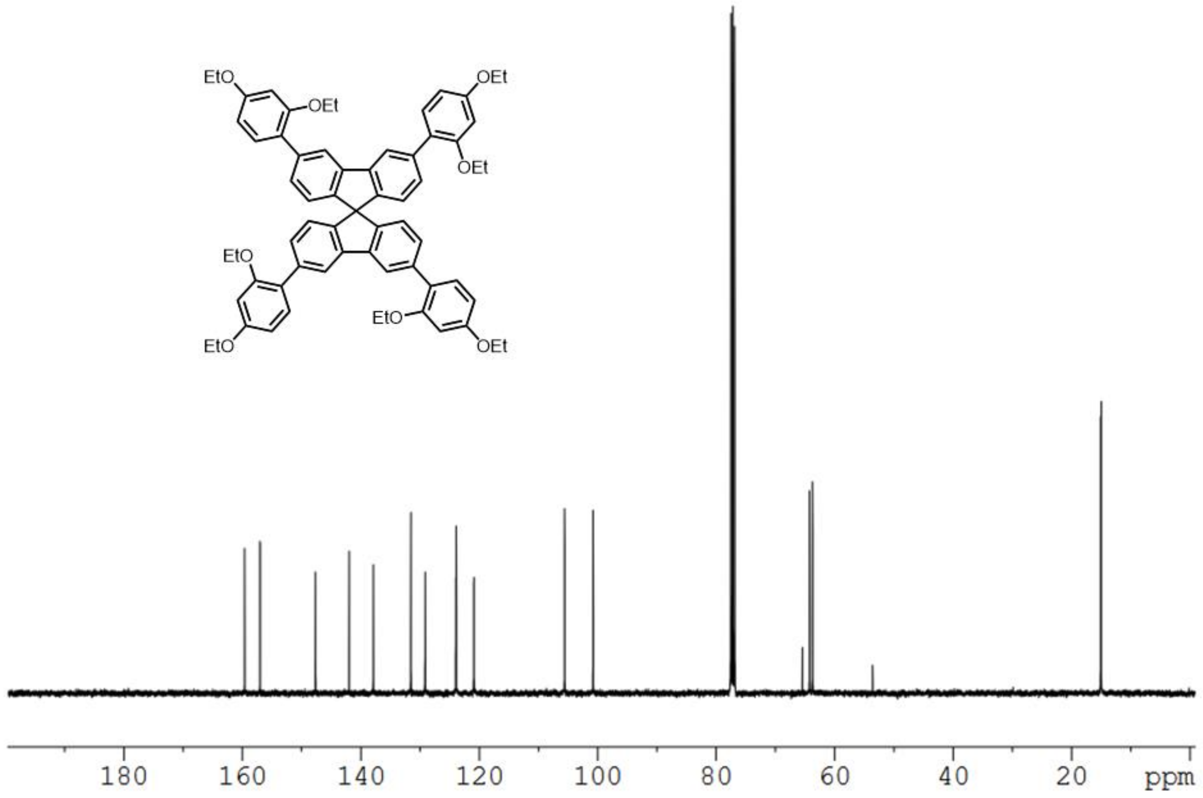

Figure S4. ^{13}C-NMR spectrum of compound **8** ($CDCl_3$, 101 MHz).

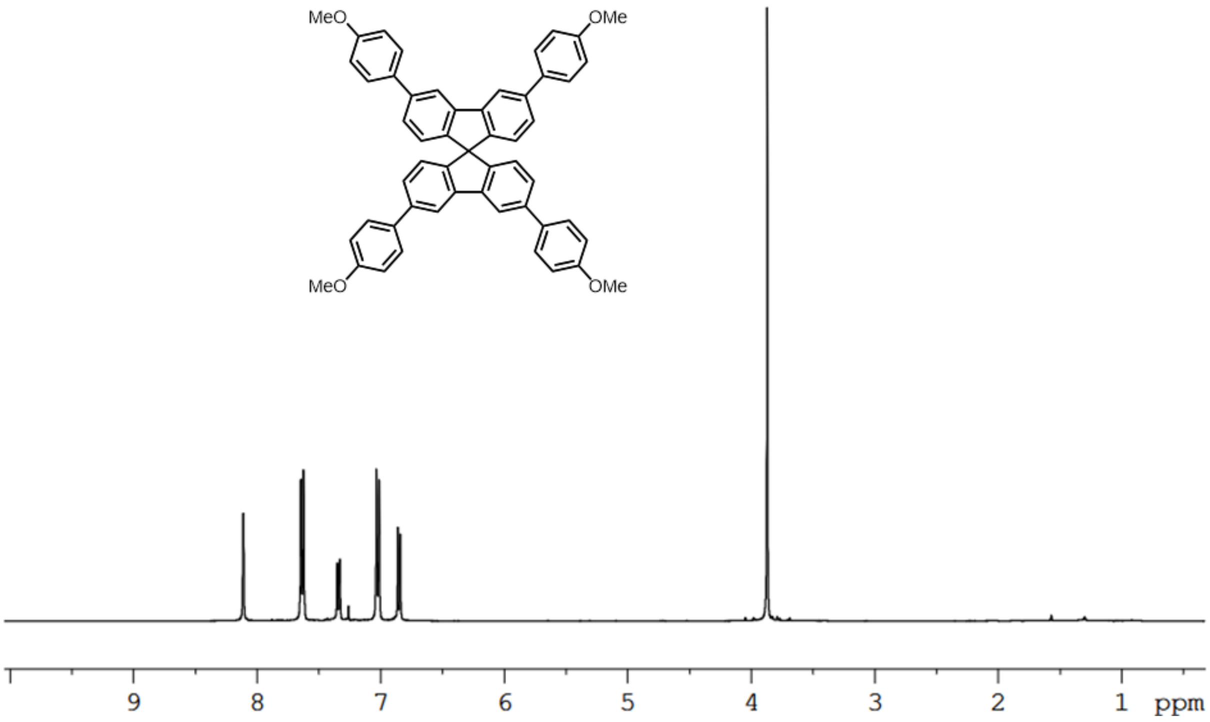

Figure S5. ^{1}H-NMR spectrum of compound **9** ($CDCl_3$, 400 MHz).

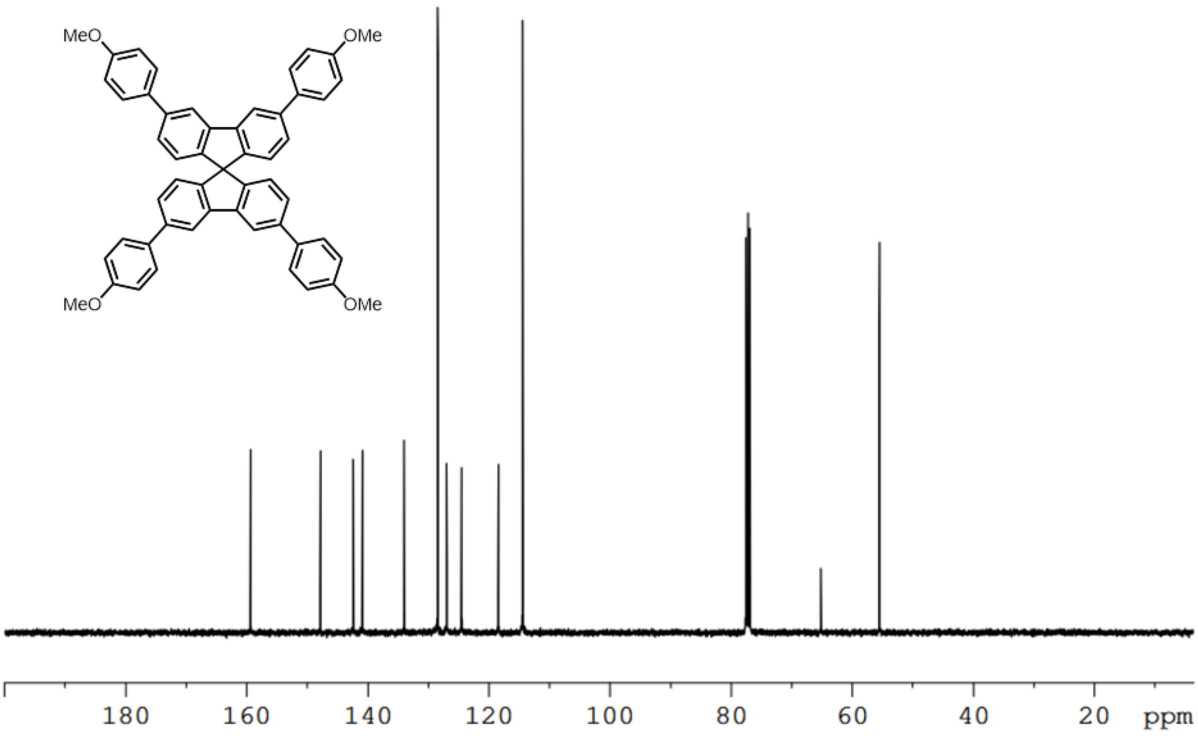

Figure S6. ^{13}C-NMR spectrum of compound **9** ($CDCl_3$, 101 MHz).

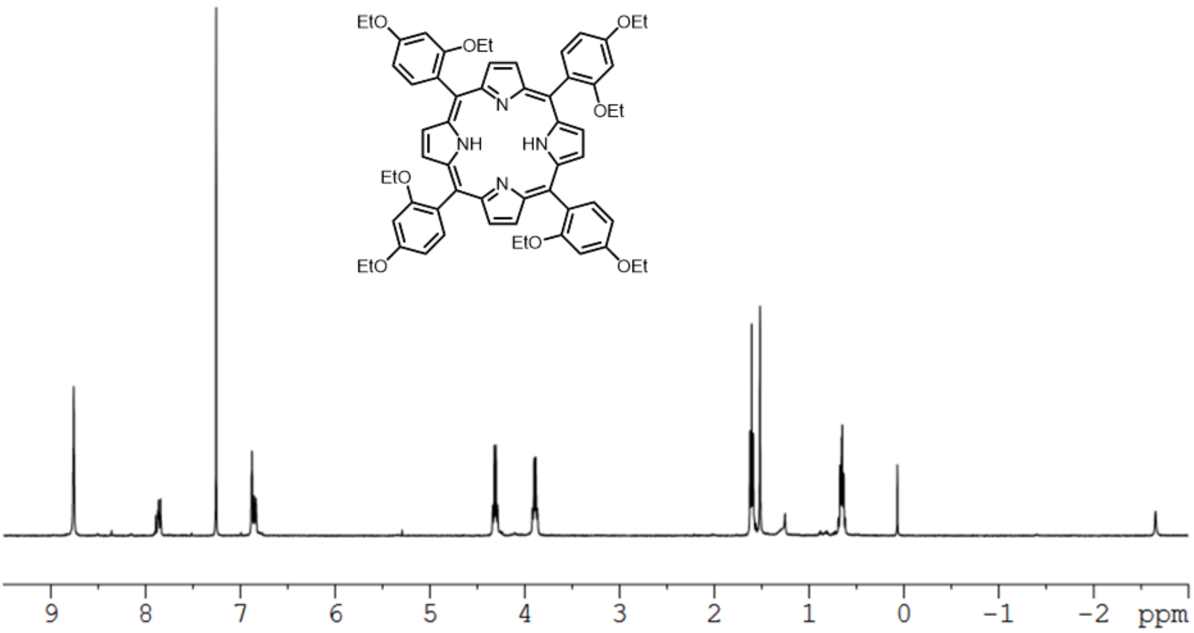

Figure S7. ^{1}H-NMR spectrum of compound **12** ($CDCl_3$, 400 MHz).

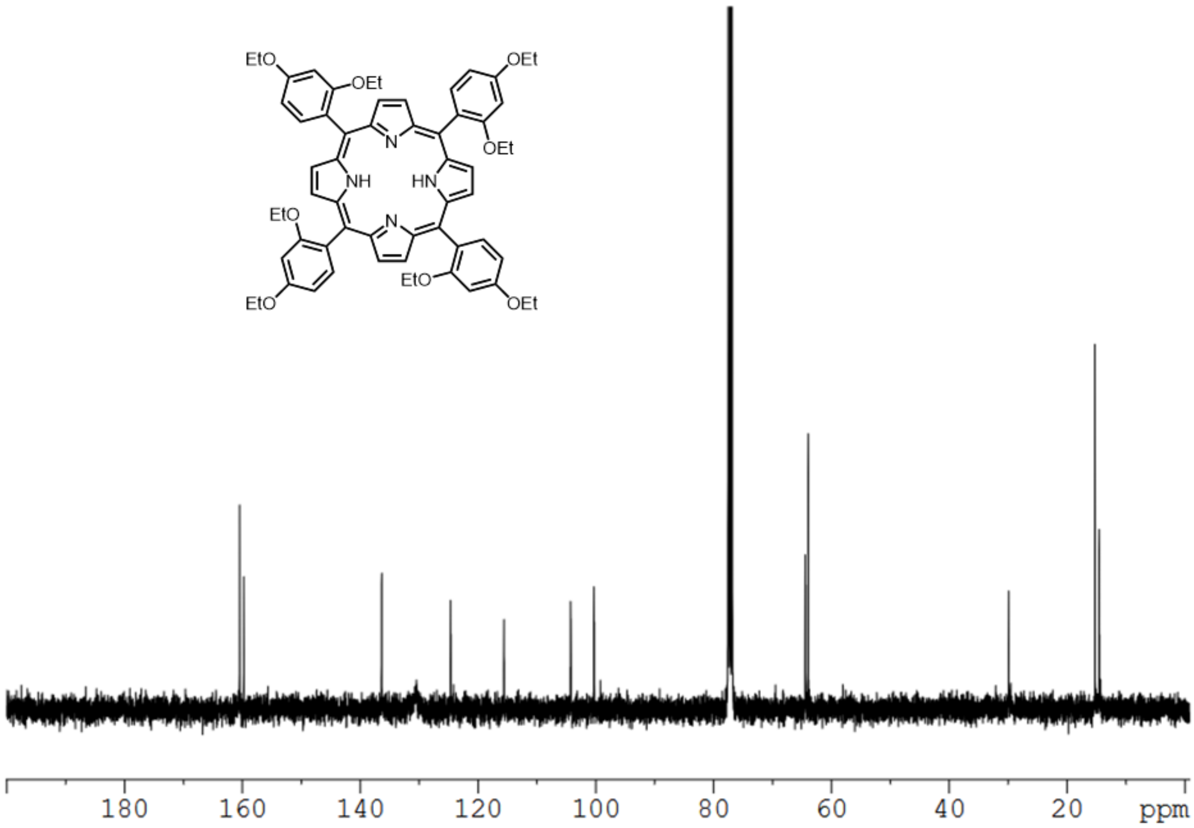

Figure S8. ^{13}C-NMR spectrum of compound **12** ($CDCl_3$, 101 MHz).

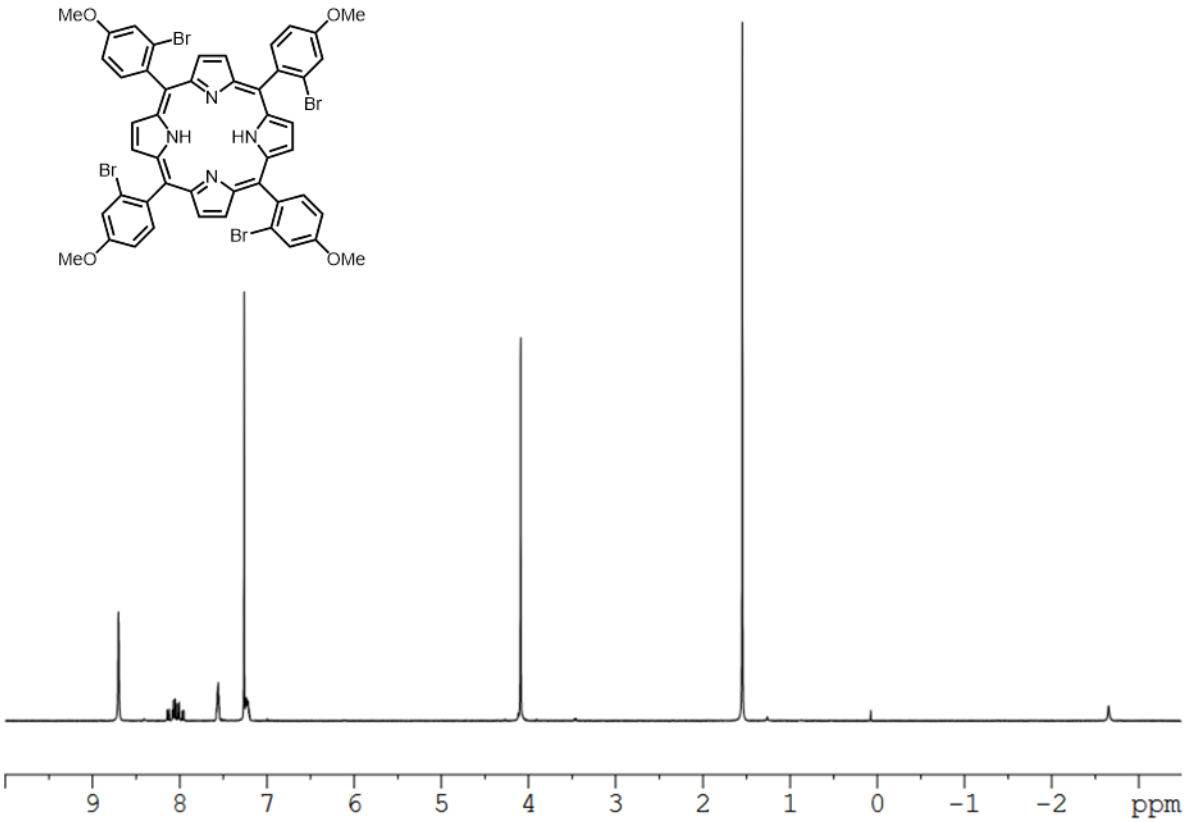

Figure S9. ^{1}H-NMR spectrum of compound **14** ($CDCl_3$, 400 MHz).

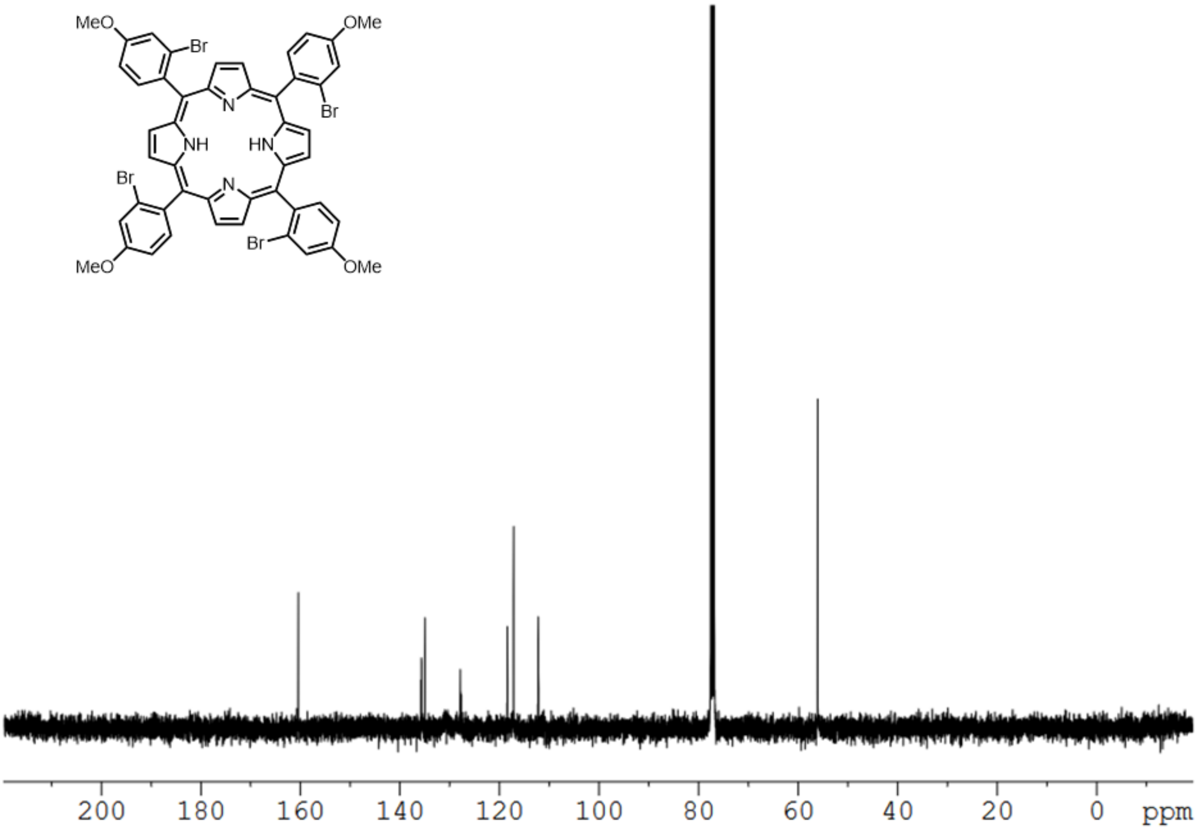

Figure S10. ^{13}C-NMR spectrum of compound **14** ($CDCl_3$, 101 MHz).

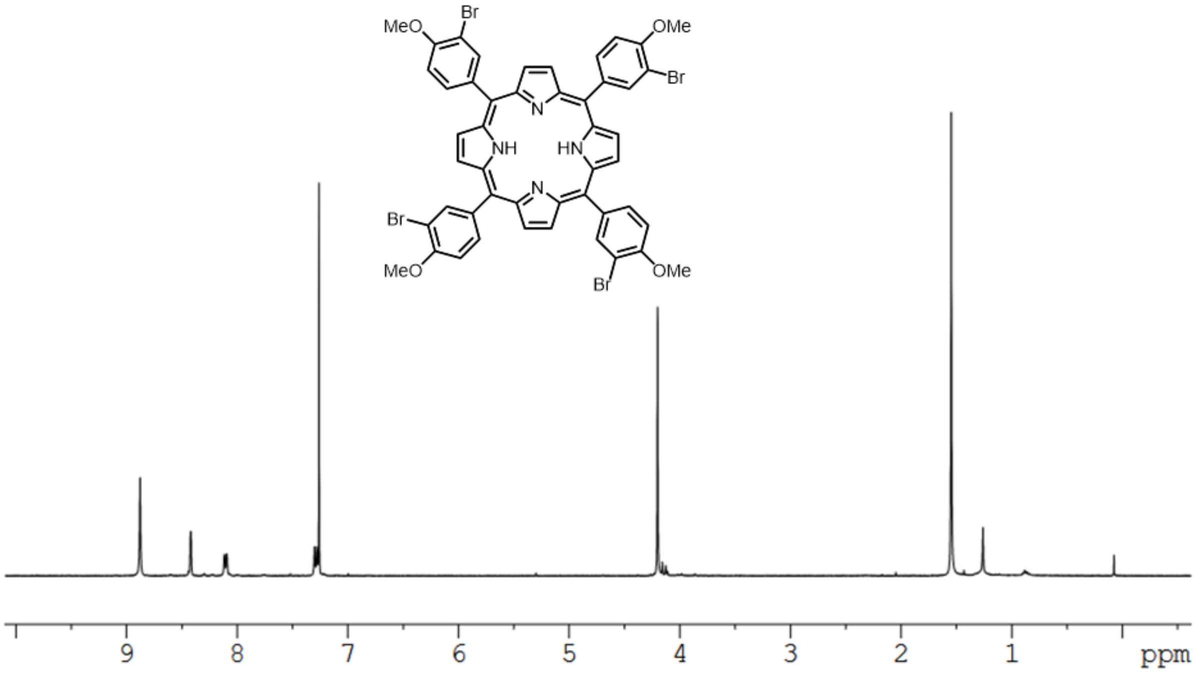

Figure S11. ^{1}H-NMR spectrum of compound **15** ($CDCl_3$, 400 MHz).

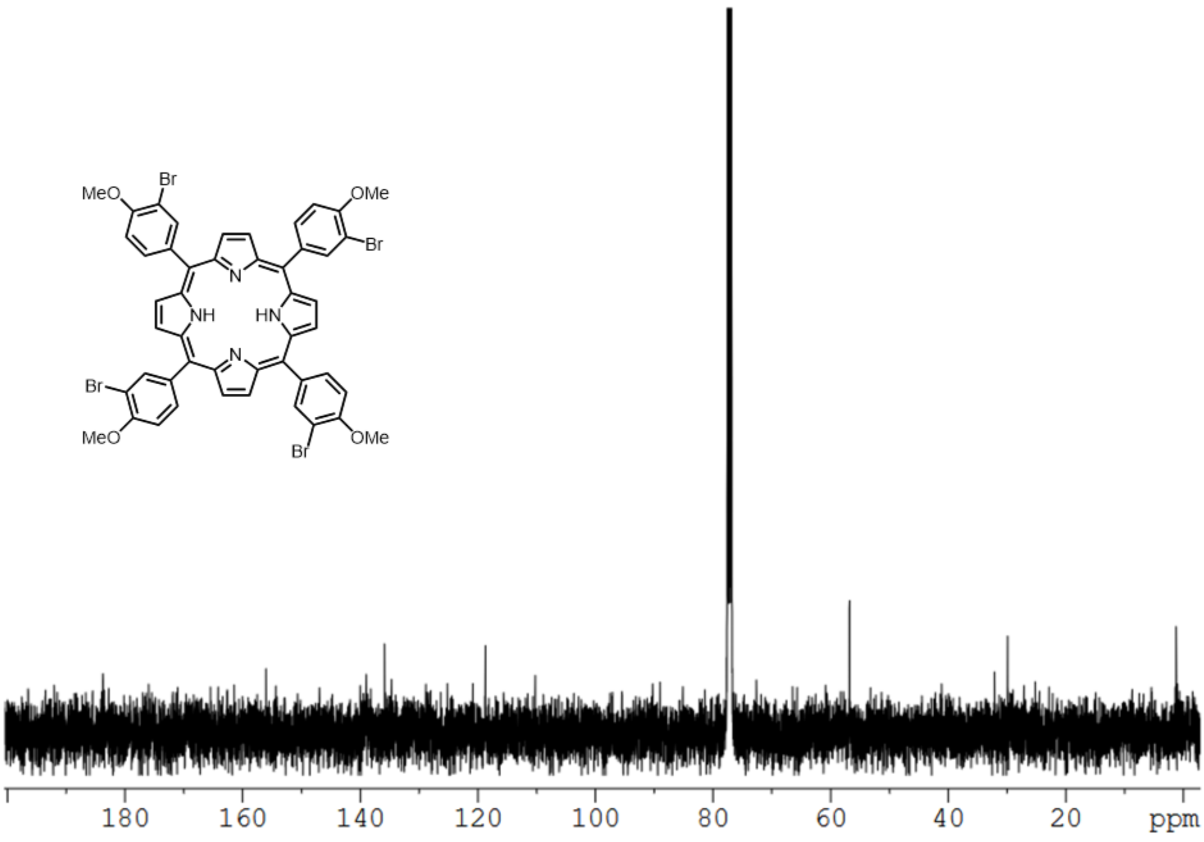

Figure S12. ^{13}C-NMR spectrum of compound **15** ($CDCl_3$, 101 MHz).

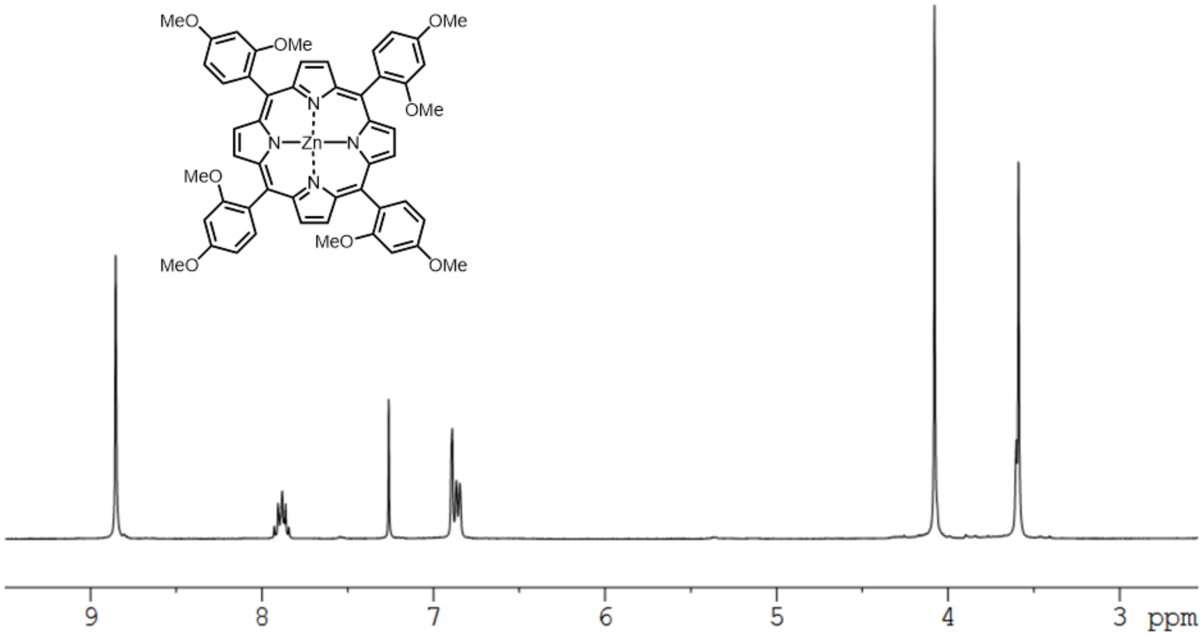

Figure S13. ^{1}H-NMR spectrum of compound **16** ($CDCl_3$, 400 MHz).

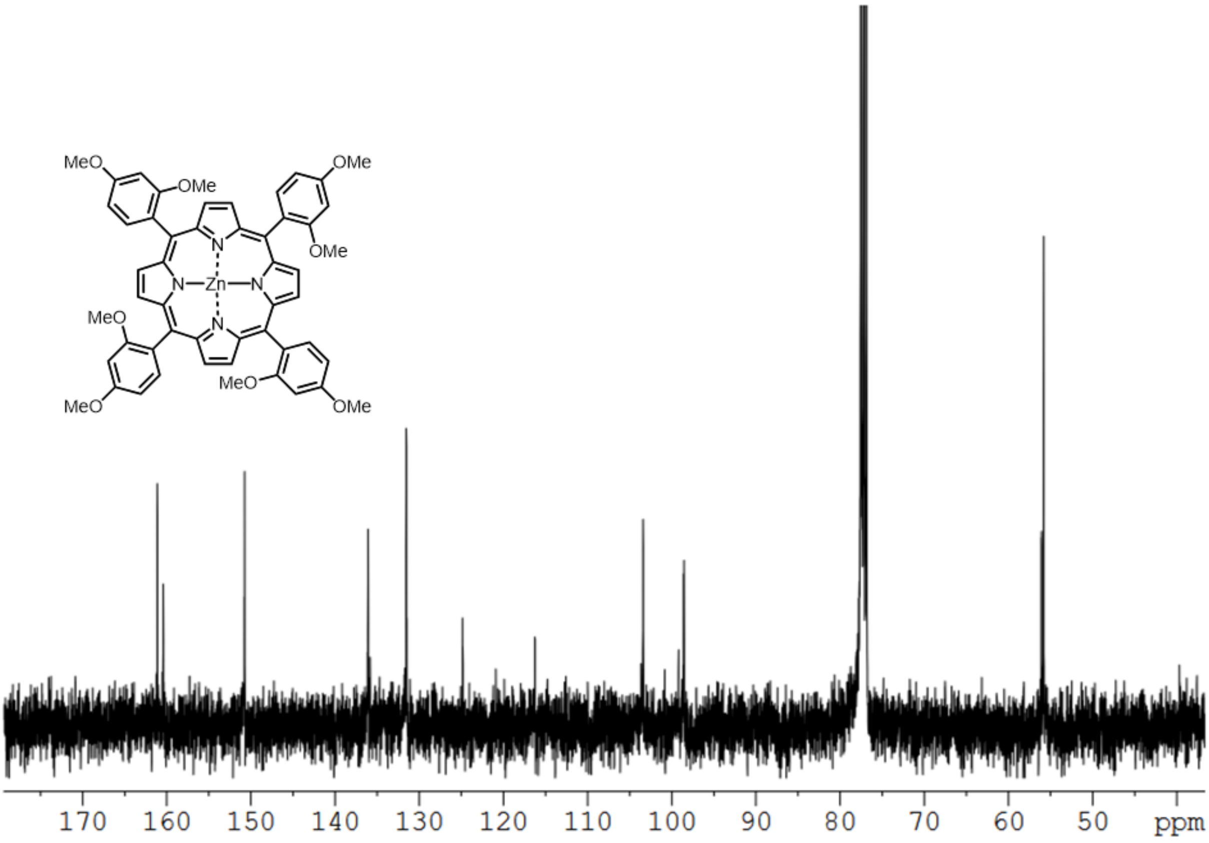

Figure S14. ^{13}C-NMR spectrum of compound **16** ($CDCl_3$, 101 MHz).

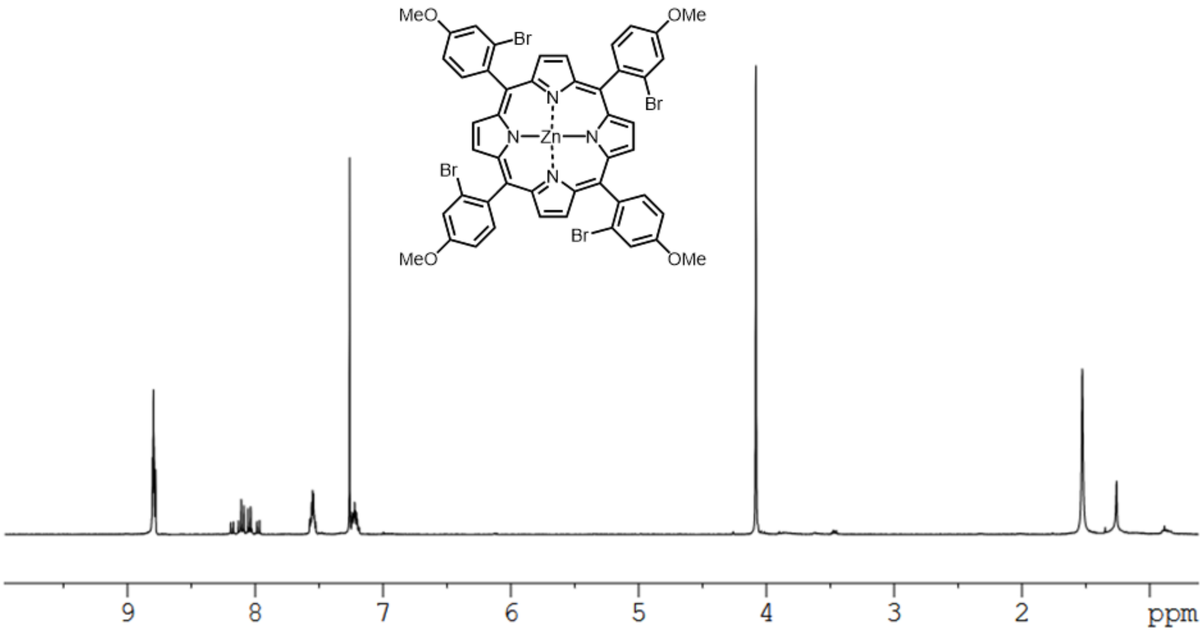

Figure S15. ^{1}H-NMR spectrum of compound **22** ($CDCl_3$, 400 MHz).

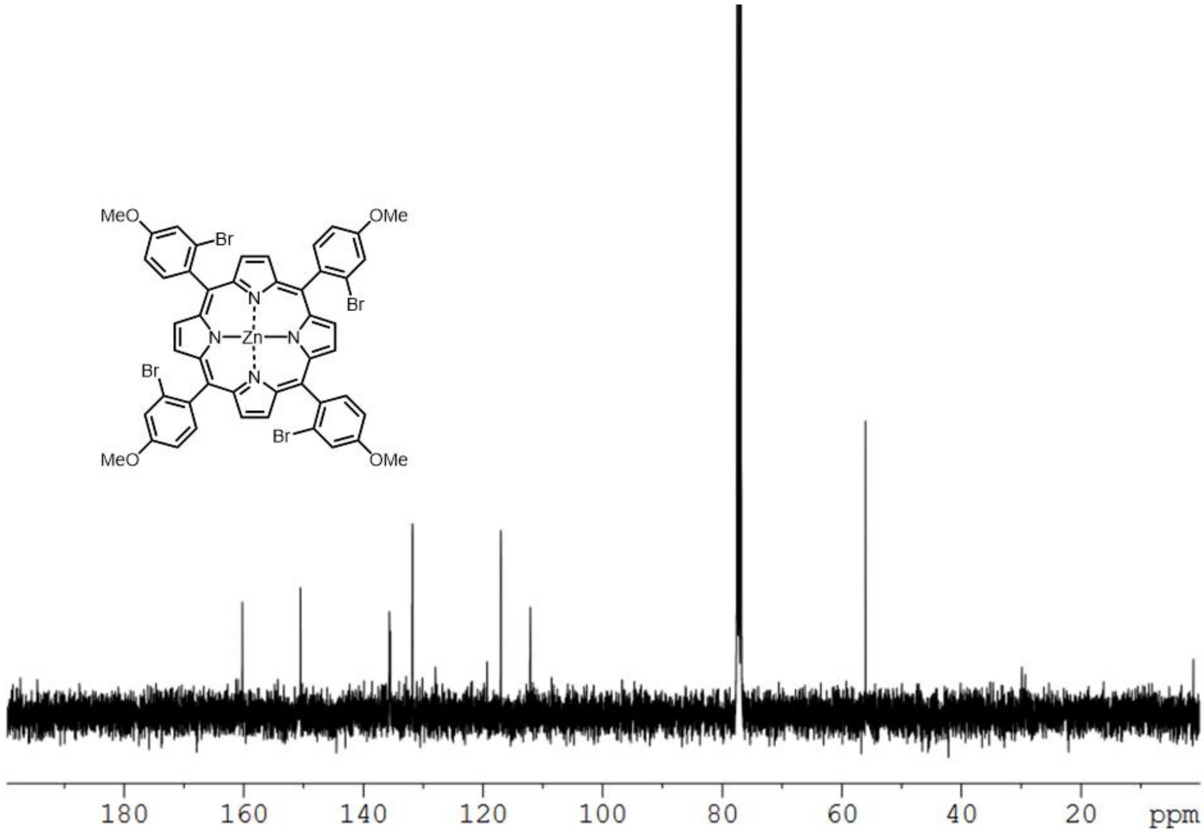

Figure S16. ^{13}C-NMR spectrum of compound **22** ($CDCl_3$, 101 MHz).

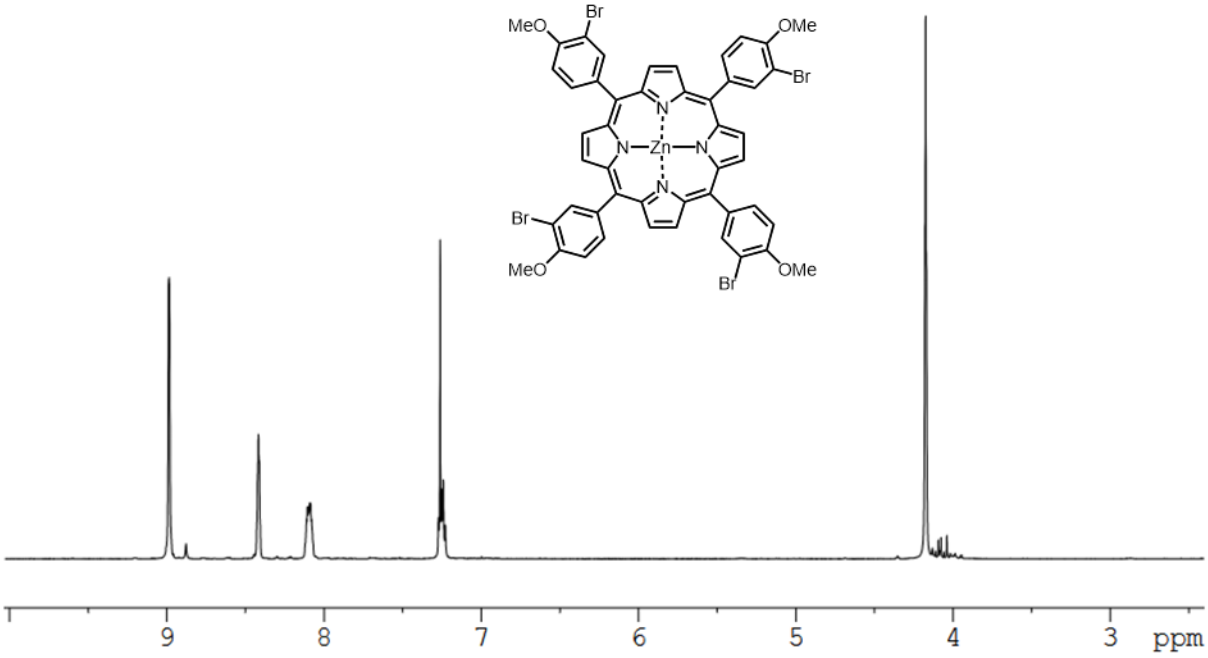

Figure S17. ^{1}H-NMR spectrum of compound **24** ($CDCl_3$, 400 MHz).

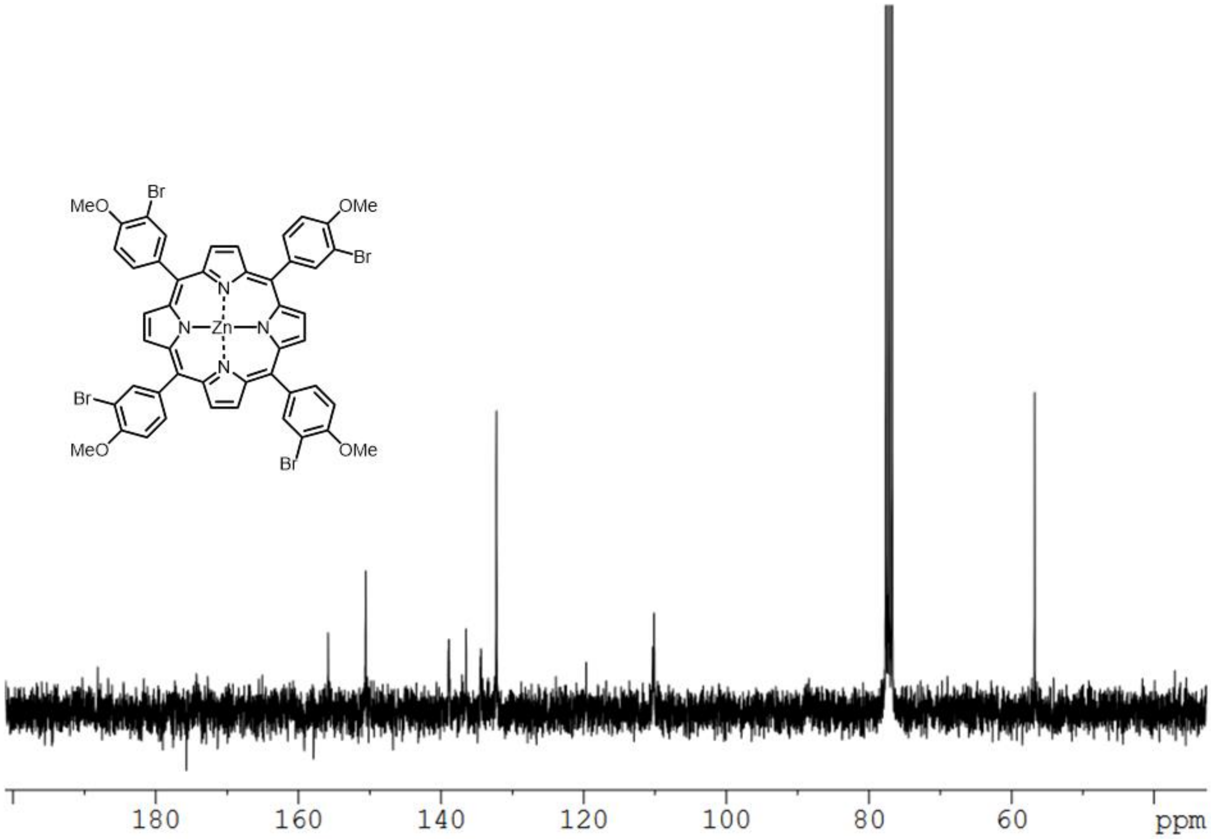

Figure S18. ^{13}C-NMR spectrum of compound **24** ($CDCl_3$, 101 MHz).

4. Crystallization Protocols

Thermal crystallization (General Protocol IV)

In a cylindrical glass vial (15 mm diameter, 40 mm height), the chaperone (2-7 mg) and liquid (20-100 μL) were heated on a pre-heated hotplate at the desired temperature (140 °C or boiling point of the liquid) until the chaperone was fully dissolved. The heating was switched off, and the solution was slowly cooled to room temperature. After up to 48 h, suitable crystals were picked and analyzed via X-ray diffraction.

Diffusion-controlled crystallization (General Protocol V)

Chaperone (2 - 7 mg) was dissolved in the liquid analyte (solvent, ca. 100 μL) in a cylindrical glass vial and placed into a bigger glass vial which contained MeOH or cyclohexane (antisolvent, 1 mL). The bigger container was closed and the antisolvent was allowed to slowly diffuse into the solvent at room temperature over no longer than 2 weeks, after which appropriate single crystals could be picked and analyzed by SC-XRD.

5. Additional Information on X-ray Crystal Structures

Solvate-free structure of 5

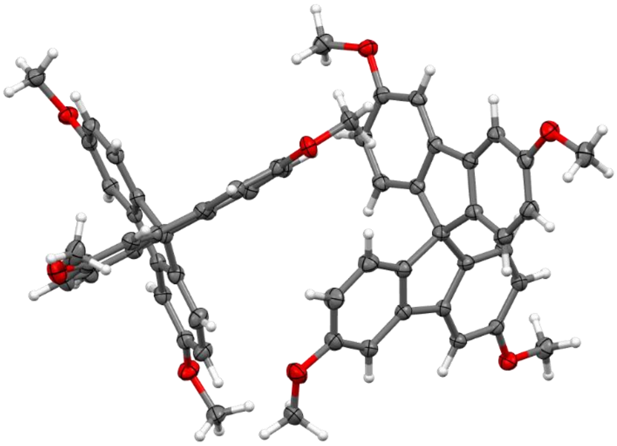

Figure S19. Crystal structure of **5** crystallized from anisole. No inclusion of guest molecules was observed. ORTEP reprsentation at 50% probability level, colors: C, grey; H, white; O, red.

Solvate structure of 7 and MeOH

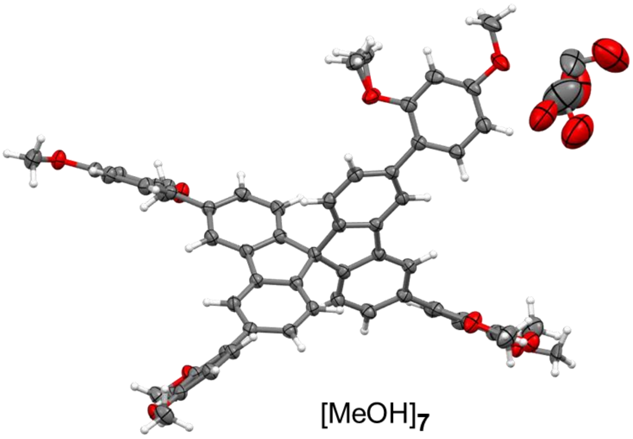

Figure S20. Solvate structure of **7** and methanol, obtained via diffusion-controlled crystallization from eugenol/MeOH. The guest is strongly disordered and parts of the methoxy groups of the host are disordered. ORTEP plots, 50% probability level, colors: C, grey; H, white; O, red.

Solvate-free structure of 8

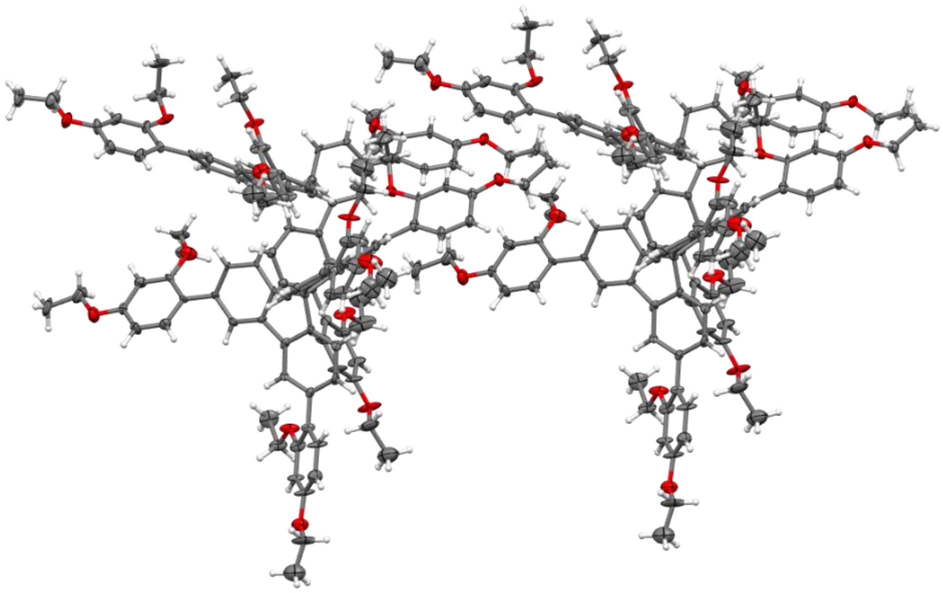

Figure S21. Solvate-free crystal structure of **8**, crystallized from eugenol/MeOH diffusion. No solvate structure was observed for this compound. ORTEP presentations at 50% probability, colors: C, grey; H, white; O, red.

Solvate structure of 24/α-(*R*)-methylbenzylamine

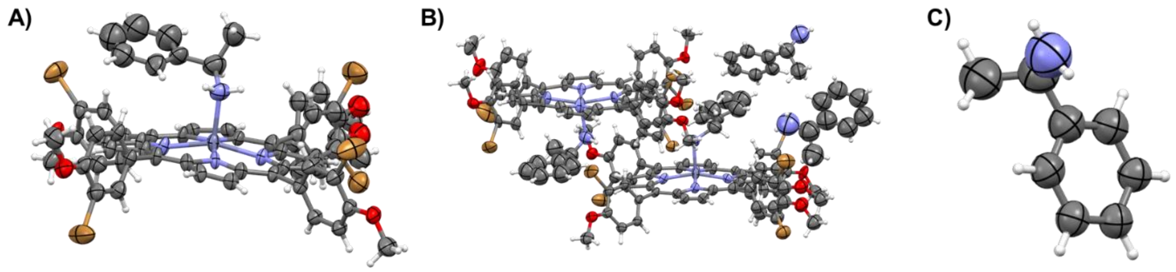

[α-(*R*)-methylbenzylamine]$_{24}$

Figure S22. Molecular structures of solvates of **24** with the chiral analyte α-(*R*)-methylbenzylamine. The chaperone shows rotational disorder in two of the four aryl moieties. A) One of the two analyte molecules that form a complex with the Zn-center of **24**. B) Asymmetric unit of the solvate structure with a molar ratio of host and guest of 2/4. C) Most well-ordered analyte molecule in the **24**/α-(*R*)-methylbenzylamine structure. The Flack-parameter is too high to allow for the determination of absolute configuration. ORTEP depiction at 50% probability level. Colors: C, grey; H, white; O, red, N, blue; Br, orange; Zn, indigo.

Structural details for the solvate-free form of 5 (CCDC 2432456)

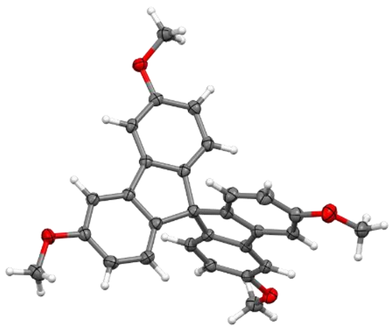

Figure S23. Asymmetric unit of the solvate-free crystal structure of **5**.

Empirical formula	$C_{29}H_{24}O_4$	
Formula weight	436.48	
Temperature	150(2) K	
Wavelength	0.71073 Å	
Crystal system, space group	Monoclinic, $P2_1/c$	
Unit cell dimensions	a = 10.3216(2) Å	α = 90°
	b = 14.7049(3) Å	β = 102.8760(10)°
	c = 14.8021(3) Å	γ = 90°
Volume	2190.14(8) $Å^3$	
Z, Calculated density	4, 1.324 g/cm3	
Absorption coefficient	0.087 mm^{-1}	
F(000)	920	
Crystal size	0.317 × 0.368 × 0.399 mm	
Theta range for data collection	1.977 to 28.314 °	
Limiting indices	$-13 \leq h \leq 13$, $-19 \leq k \leq 11$, $-19 \leq l \leq 19$	
Reflections collected/ unique	20276/5440 [R(int) = 0.0308]	
Completeness to theta = 25.242	99.9 %	
Absorption correction	Multi-scan	
Max. and min. transmission	0.7457 and 0.7135	
Refinement method	Full-matrix least-squares on F^2	
Data/restraints/parameters	5440/0/303	
Goodness-of-fit on F^2	1.031	
Final R indices [I>2sigma(I)]	R1 = 0.0569, wR2 = 0.1055	
R indices (all data)	R1 = 0.0408, wR2 = 0.0958	
Extinction coefficient	0.0082(8)	
Largest diff. peak and hole	0.299 and -0.193 e.$Å^{-3}$	

Structural details for the solvate form of 7 and anisole (CCDC 2432455)

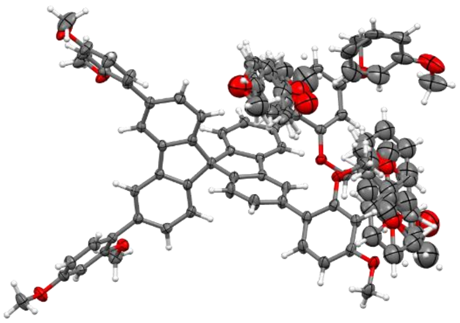

Figure S24. Asymmetric unit of the **7**/anisole crystal structure.

Empirical formula	$C_{67.5}H_{60}O_{9.5}$	
Formula weight	1023.15	
Temperature	150(2) K	
Wavelength	0.71073 Å	
Crystal system, space group	Triclinic, P-1	
Unit cell dimensions	a = 12.5153(6) Å	α = 61.126(2)°
	b = 15.7166(8) Å	β = 84.277(3)°
	c = 16.2521(9) Å	γ = 85.785(3)°
Volume	2784.2(3) Å^3	
Z, Calculated density	2, 1.220 g/cm3	
Absorption coefficient	0.081 mm^{-1}	
F(000)	1082	
Crystal size	0.072 × 0.186 × 0.345 mm	
Theta range for data collection	1.435 to 25.410 °	
Limiting indices	-15 ≤ h ≤ 15, -18 ≤ k ≤ 17, -19 ≤ l ≤ 19	
Reflections collected/ unique	10173/6298 [R(int) = 0.0683]	
Completeness to theta = 25.242	99.4 %	
Absorption correction	Multi-scan	
Max. and min. transmission	0.7452 and 0.6958	
Refinement method	Full-matrix least-squares on F^2	
Data/restraints/parameters	10173/459/877	
Goodness-of-fit on F^2	1.045	
Final R indices [I>2sigma(I)]	R1 = 0.1183, wR2 = 0.1621	
R indices (all data)	R1 = 0.0688, wR2 = 0.1858	
Extinction coefficient	0.0041(9)	
Largest diff. peak and hole	0.733 and -0.387 e.Å^{-3}	

Structural details for the solvate form of 7 and *p*-xylene (CCDC 2432453)

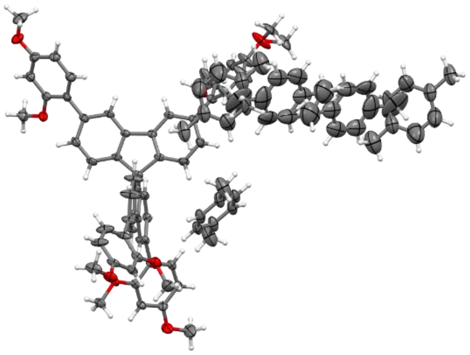

Figure S25. Asymmetric unit of the **7**/*p*-xylene crystal structure.

Empirical formula	$C_{69}H_{63}O_8$	
Formula weight	1020.19	
Temperature	150(2) K	
Wavelength	0.71073 Å	
Crystal system, space group	Triclinic, P-1	
Unit cell dimensions	a = 12.4742(7) Å	α = 61.567(2)°
	b = 15.6866(8) Å	β = 83.869(2)°
	c = 16.2037(9) Å	γ = 85.776(2)°
Volume	2771.3(3) $Å^3$	
Z, Calculated density	2, 1.223 g/cm3	
Absorption coefficient	0.079 mm^{-1}	
F(000)	1082	
Crystal size	0.045 × 0.170 × 0.210 mm	
Theta range for data collection	1.434 to 25.333 °	
Limiting indices	$-15 \leq h \leq 10$, $-18 \leq k \leq 17$, $-19 \leq l \leq 19$	
Reflections collected/ unique	10070/5640 [R(int) = 0.0575]	
Completeness to theta = 25.242	99.5 %	
Absorption correction	Multi-scan	
Max. and min. transmission	0.7452 and 0.7193	
Refinement method	Full-matrix least-squares on F^2	
Data/restraints/parameters	10070/128/762	
Goodness-of-fit on F^2	1.002	
Final R indices [I>2sigma(I)]	R1 = 0.1262, wR2 = 0.1422	
R indices (all data)	R1 = 0.0560, wR2 = 0.1165	
Extinction coefficient	0.0047(7)	
Largest diff. peak and hole	0.318 and -0.259 e.$Å^{-3}$	

Structural details for the solvate form of 7 and methanol (CCDC 2432457)

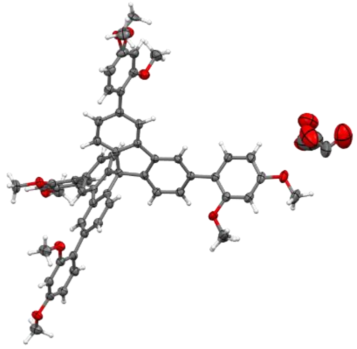

Figure S26. Asymmetric unit of the **7**/methanol crystal structure.

Empirical formula	$C_{58}H_{48}O_9$	
Formula weight	888.96	
Temperature	150(2) K	
Wavelength	0.71073 Å	
Crystal system, space group	Triclinic, P-1	
Unit cell dimensions	a = 12.3164(6) Å	α = 74.533(2)°
	b = 14.1303(8) Å	β = 67.108(2)°
	c = 15.3021(8) Å	γ = 73.516(2)°
Volume	2315.4(2) $Å^3$	
Z, Calculated density	2, 1.275 g/cm3	
Absorption coefficient	0.085 mm^{-1}	
F(000)	936	
Crystal size	0.180 × 0.260 × 0.530 mm	
Theta range for data collection	2.613 to 25.394 °	
Limiting indices	$-14 \leq h \leq 14$, $-16 \leq k \leq 17$, $-18 \leq l \leq 18$	
Reflections collected/ unique	8416/5633 [R(int) = 0.0377]	
Completeness to theta = 25.242	99.1 %	
Absorption correction	Multi-scan	
Max. and min. transmission	0.7452 and 0.6952	
Refinement method	Full-matrix least-squares on F^2	
Data/restraints/parameters	8416/84/676	
Goodness-of-fit on F^2	1.032	
Final R indices [I>2sigma(I)]	R1 = 0.0788, wR2 = 0.0995	
R indices (all data)	R1 = 0.0446, wR2 = 0.0904	
Extinction coefficient	0.0026(4)	
Largest diff. peak and hole	0.284 and -0.207 e.$Å^{-3}$	

Structural details for the solvate-free form of 8 (CCDC 2432458)

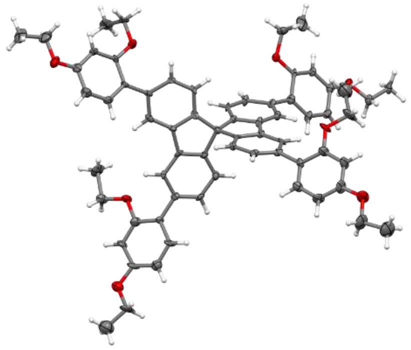

Figure S27. Asymmetric unit of the solvate-free crystal structure of **8**. Measured crystal presented twinning.

Empirical formula	$C_{65}H_{64}O_8$	
Formula weight	973.16	
Temperature	150(2) K	
Wavelength	0.71073 Å	
Crystal system, space group	Monoclinic, $P2_1/c$	
Unit cell dimensions	a = 15.969(4) Å	α = 90°
	b = 26.604(8) Å	β = 92.496(10)°
	c = 12.875(4) Å	γ = 90°
Volume	5465(3) $Å^3$	
Z, Calculated density	4, 1.183 g/cm3	
Absorption coefficient	0.077 mm^{-1}	
F(000)	2072	
Crystal size	0.084 × 0.090 × 0.664 mm	
Theta range for data collection	1.488 to 25.000 °	
Limiting indices	$-16 \leq h \leq 18$, $-25 \leq k \leq 31$, $-15 \leq l \leq 14$	
Reflections collected/ unique	9624/4853 [R(int) = 0.1171]	
Completeness to theta = 25.000	99.8 %	
Absorption correction	Multi-scan	
Max. and min. transmission	0.8620 and 0.7554	
Refinement method	Full-matrix least-squares on F^2	
Data/restraints/parameters	9624/72/659	
Goodness-of-fit on F^2	1.058	
Final R indices [I>2sigma(I)]	R1 = 0.2616, wR2 = 0.3859	
R indices (all data)	R1 = 0.1547, wR2 = 0.3467	
Extinction coefficient	-	
Largest diff. peak and hole	0.752 and -0.539 e.$Å^{-3}$	

Structural details for the solvate form of 9 and toluene (CCDC 2432450)

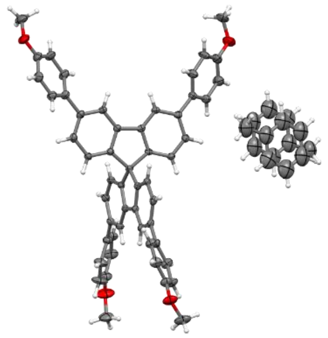

Figure S28. Asymmetric unit of the **9**/toluene crystal structure.

Empirical formula	$C_{60}H_{48}O_4$	
Formula weight	832.98	
Temperature	145(2) K	
Wavelength	0.71073 Å	
Crystal system, space group	Orthorhombic, Pccn	
Unit cell dimensions	a = 15.6780(7) Å	α = 90°
	b = 24.7946(13) Å	β = 90°
	c = 11.4904(6) Å	γ = 90°
Volume	4466.7(4) $Å^3$	
Z, Calculated density	4, 1.239 g/cm3	
Absorption coefficient	0.076 mm^{-1}	
F(000)	1760	
Crystal size	0.060 × 0.130 × 0.520 mm	
Theta range for data collection	1.537 to 25.390 °	
Limiting indices	$-18 \le h \le 18$, $-29 \le k \le 29$, $-13 \le l \le 13$	
Reflections collected/ unique	4105/2426 [R(int) = 0.0902]	
Completeness to theta = 25.242	100.0 %	
Absorption correction	Multi-scan	
Max. and min. transmission	0.7452 and 0.6108	
Refinement method	Full-matrix least-squares on F^2	
Data/restraints/parameters	4105/127/324	
Goodness-of-fit on F^2	1.035	
Final R indices [I>2sigma(I)]	R1 = 0.1135, wR2 = 0.1326	
R indices (all data)	R1 = 0.0532, wR2 = 0.1109	
Extinction coefficient	0.0030(4)	
Largest diff. peak and hole	0.291 and -0.357 e.$Å^{-3}$	

Structural details for the solvate form of 14 and toluene (CCDC 2432451)

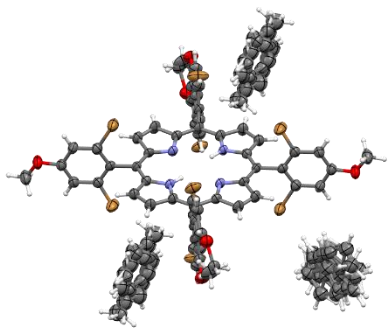

Figure S29. Asymmetric unit of the **14**/toluene crystal structure.

Empirical formula	$C_{62}H_{48}Br_4N_4O_4$	
Formula weight	1232.68	
Temperature	145(2) K	
Wavelength	0.71073 Å	
Crystal system, space group	Monoclinic, $P2_1/c$	
Unit cell dimensions	a = 12.4751(6) Å	α = 90°
	b = 15.9369(7) Å	β = 105.253(3)°
	c = 14.0831(6) Å	γ = 90°
Volume	2701.3(2) Å^3	
Z, Calculated density	2, 1.516 g/cm3	
Absorption coefficient	3.032 mm^{-1}	
F(000)	1240	
Crystal size	0.035 × 0.229 × 0.310 mm	
Theta range for data collection	1.692 to 25.384 °	
Limiting indices	-11 ≤ h ≤ 15, -19 ≤ k ≤ 19, -16 ≤ l ≤ 16	
Reflections collected/ unique	4928/3013 [R(int) = 0.0464]	
Completeness to theta = 25.242	99.6 %	
Absorption correction	Multi-scan	
Max. and min. transmission	0.9452 and 0.5406	
Refinement method	Full-matrix least-squares on F^2	
Data/restraints/parameters	4928/732/615	
Goodness-of-fit on F^2	1.031	
Final R indices [I>2sigma(I)]	R1 = 0.1078, wR2 = 0.1487	
R indices (all data)	R1 = 0.0551, wR2 = 0.1283	
Extinction coefficient	-	
Largest diff. peak and hole	0.725 and -0.439 e.Å^{-3}	

Structural details for the solvate form of 24 and α-(*R*)-methylbenzylamine (CCDC 2432452)

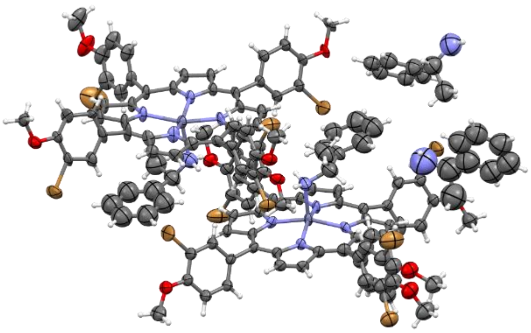

Figure S30. Asymmetric unit of the **24**/α-(*R*)-methylbenzylamine crystal structure.

Empirical formula	$C_{60}H_{48.5}Br_4N_{5.5}O_4Zn$	
Formula weight	1295.55	
Temperature	150(2) K	
Wavelength	1.54178 Å	
Crystal system, space group	Triclinic, P1	
Unit cell dimensions	a = 14.4391(16) Å	α = 92.030(8)°
	b = 14.4731(16) Å	β = 113.409(7)°
	c = 15.9367(17) Å	γ = 104.454(7)°
Volume	2925.4(6) $Å^3$	
Z, Calculated density	2, 1.471 g/cm3	
Absorption coefficient	4.176 mm^{-1}	
F(000)	1298	
Crystal size	0.048 × 0.078 × 0.220 mm	
Theta range for data collection	3.482 to 65.590 °	
Limiting indices	-17 ≤ h ≤ 16, -16 ≤ k ≤ 16, -18 ≤ l ≤ 18	
Reflections collected/ unique	14951/9812 [R(int) = 0.0581]	
Completeness to theta = 65.590	97.1 %	
Absorption correction	Multi-scan	
Max. and min. transmission	0.8604 and 0.5602	
Refinement method	Full-matrix least-squares on F^2	
Data/restraints/parameters	14951/1704/1477	
Goodness-of-fit on F^2	1.046	
Final R indices [I>2sigma(I)]	R1 = 0.1136, wR2 = 0.1881	
R indices (all data)	R1 = 0.0722, wR2 = 0.1645	
Extinction coefficient	-	
Largest diff. peak and hole	1.504 and -0.994 e.$Å^{-3}$	

Representative Hirshfeld Fingerprint Plots

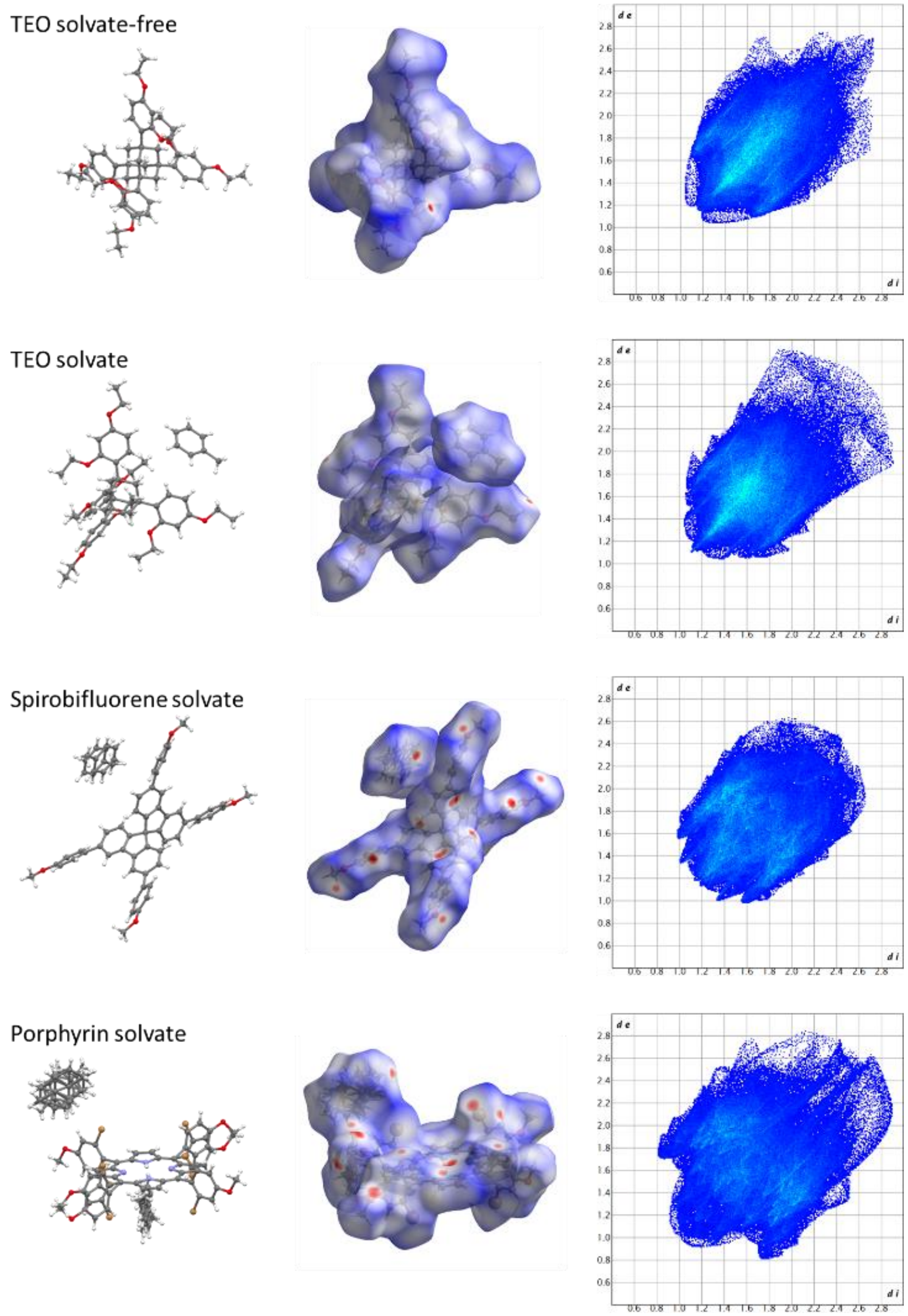

Figure S31. Hirshfeld surface 2D fingerprint plots[S15] for representative structures from either of the three classes of chaperone candidates with toluene as guest molecule. The underlying structures can be found in CCDC entries 1521509, 1918377, 2432450 and 2432451.

Overlay of Conformations in Crystal Structures for Porphyrin and Spirobifluorenes

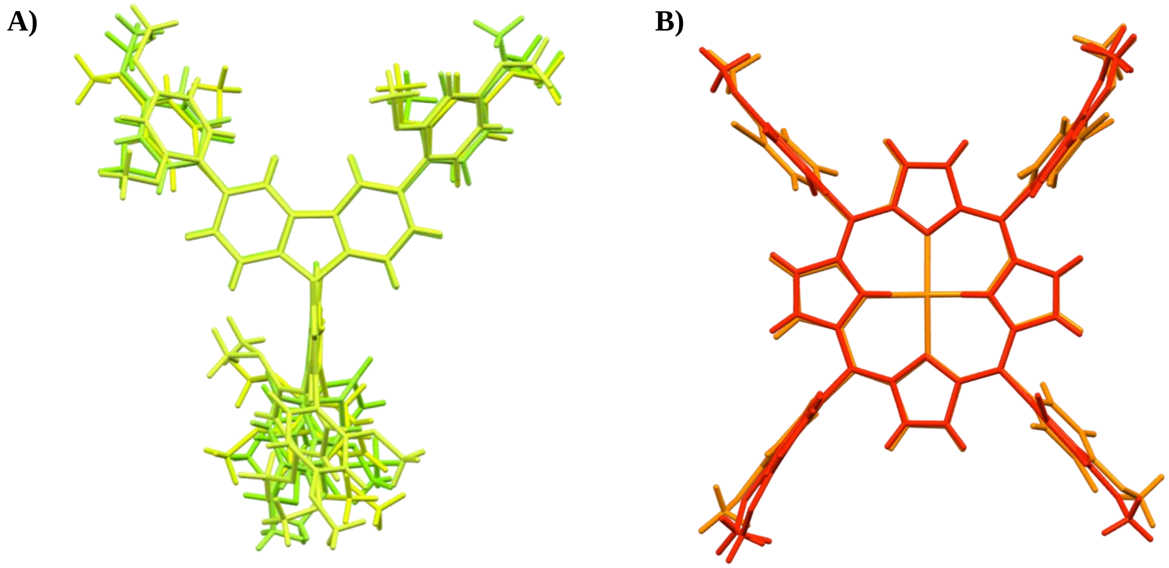

Figure S32. Overlay of conformers of non-TAA chaperone candidates in crystal structures reported here. A) Overlay of the conformations in the four TAS solvate structures, with three from compound **7** and one from compound **9**. B) Overlay of the TPPs in solvate structures obtained with **14** and **24**. See Table 1 of the main paper for the details of the structures underlying these overlays. The overlays are analogous to what is presented for TAAs in Figure 6 of the main paper, except that fewer structures are available for these compounds. We note that in either case, two different molecules are being plotted here, making the conformations look more diverse than in the 'single compound overlays' of Figure 6.

6. Theoretical Work

The following tables list the numerical values underlying the plots shown in Figure 7.

Table S3. Dihedral angles of the model compound *ortho*-bromophenyladamantane and their calculated final Gibbs free energy at 293.15 K.

Angle (rel. to staggered) [°]	Energy (abs.) [Eh]	Energy (rel. to staggered) [Eh]	Energy (rel. to ideal conformation) [kJ/mol]
0	-3193,74615116	-3193,74615116	-8385180,52
10	-3193,74519005	-3193,74519005	-8385178,00
20	-3193,74735938	-3193,74735938	-8385183,69
30	-3193,74734928	-3193,74734928	-8385183,67
40	-3193,74724863	-3193,74724863	-8385183,40
50	-3193,74538252	-3193,74538252	-8385178,50
60	-3193,74603157	-3193,74603157	-8385180,21
70	-3193,74856366	-3193,74856366	-8385186,85
80	-3193,75219509	-3193,75219509	-8385196,39
90	-3193,75356593	-3193,75356593	-8385199,99
100	-3193,75284075	-3193,75284075	-8385198,08
110	-3193,75070900	-3193,75070900	-8385192,49
120	-3193,74615116	-3193,74615116	-8385180,52
130	-3193,74519005	-3193,74519005	-8385178,00
140	-3193,74735938	-3193,74735938	-8385183,69
150	-3193,74734928	-3193,74734928	-8385183,67
160	-3193,74724863	-3193,74724863	-8385183,40
170	-3193,74538252	-3193,74538252	-8385178,50
180	-3193,74603157	-3193,74603157	-8385180,21
190	-3193,74856366	-3193,74856366	-8385186,85
200	-3193,75219509	-3193,75219509	-8385196,39
210	-3193,75356593	-3193,75356593	-8385199,99
220	-3193,75284075	-3193,75284075	-8385198,08
230	-3193,75070900	-3193,75070900	-8385192,49
240	-3193,74615116	-3193,74615116	-8385180,52
250	-3193,74519005	-3193,74519005	-8385178,00
260	-3193,74735938	-3193,74735938	-8385183,69
270	-3193,74734928	-3193,74734928	-8385183,67
280	-3193,74724863	-3193,74724863	-8385183,40
290	-3193,74538252	-3193,74538252	-8385178,50
300	-3193,74603157	-3193,74603157	-8385180,21
310	-3193,74856366	-3193,74856366	-8385186,85
320	-3193,75219509	-3193,75219509	-8385196,39
330	-3193,75356593	-3193,75356593	-8385199,99
340	-3193,75284075	-3193,75284075	-8385198,08
350	-3193,75070900	-3193,75070900	-8385192,49

Table S4. Dihedral angles of the model compound *ortho*-methoxyphenyladamantane and their calculated final Gibbs free energy at 293.15 K.

Angle (rel. To staggered) [°]	Energy (abs.) [Eh]	Energy (rel. to staggered) [Eh]	Energy (rel. to ideal conformation) [kJ/mol]
0	-734,9573177	-734,95731770	-1929630,44
10	-734,9566477	-734,95664772	-1929628,68
20	-734,9583509	-734,95835087	-1929633,15
30	-734,9587368	-734,95873681	-1929634,16
40	-734,9574202	-734,95742017	-1929630,71
50	-734,9570660	-734,95706603	-1929629,78
60	-734,9579821	-734,95798213	-1929632,18
70	-734,9603641	-734,96036408	-1929638,44
80	-734,9635221	-734,96352207	-1929646,73
90	-734,9643205	-734,96432053	-1929648,82
100	-734,9630406	-734,96304059	-1929645,46
110	-734,9595664	-734,95956642	-1929636,34
120	-734,9573177	-734,95731770	-1929630,44
130	-734,9566477	-734,95664772	-1929628,68
140	-734,9583509	-734,95835087	-1929633,15
150	-734,9587368	-734,95873681	-1929634,16
160	-734,9574202	-734,95742017	-1929630,71
170	-734,9570660	-734,95706603	-1929629,78
180	-734,9579821	-734,95798213	-1929632,18
190	-734,9603641	-734,96036408	-1929638,44
200	-734,9635221	-734,96352207	-1929646,73
210	-734,9643205	-734,96432053	-1929648,82
220	-734,9630406	-734,96304059	-1929645,46
230	-734,9595664	-734,95956642	-1929636,34
240	-734,9573177	-734,95731770	-1929630,44
250	-734,9566477	-734,95664772	-1929628,68
260	-734,9583509	-734,95835087	-1929633,15
270	-734,9587368	-734,95873681	-1929634,16
280	-734,9574202	-734,95742017	-1929630,71
290	-734,9570660	-734,95706603	-1929629,78
300	-734,9579821	-734,95798213	-1929632,18
310	-734,9603641	-734,96036408	-1929638,44
320	-734,9635221	-734,96352207	-1929646,73
330	-734,9643205	-734,96432053	-1929648,82
340	-734,9630406	-734,96304059	-1929645,46
350	-734,9595664	-734,95956642	-1929636,34

Table S5. Dihedral angles of the model compound *ortho*-fluorophenyladamantane and their calculated final Gibbs free energy at 293.15 K.

Angle (rel.to staggered) [°]	Energy (abs.) [Eh]	Energy (rel. to staggered) [Eh]	Energy (rel. to ideal conformation) [kJ/mol]
0	-719,7513119	-719,75131193	-1889707,07
10	-719,7509663	-719,75096630	-1889706,16
20	-719,7527893	-719,75278929	-1889710,95
30	-719,7529269	-719,75292688	-1889711,31
40	-719,7533853	-719,75338526	-1889712,51
50	-719,7512849	-719,75128486	-1889707,00
60	-719,7518582	-719,75185816	-1889708,50
70	-719,7536741	-719,75367412	-1889713,27
80	-719,7564269	-719,75642687	-1889720,50
90	-719,7571034	-719,75710337	-1889722,27
100	-719,7560213	-719,75602127	-1889719,43
110	-719,7529993	-719,75299928	-1889711,50
120	-719,7513119	-719,75131193	-1889707,07
130	-719,7509663	-719,75096630	-1889706,16
140	-719,7527893	-719,75278929	-1889710,95
150	-719,7529269	-719,75292688	-1889711,31
160	-719,7533853	-719,75338526	-1889712,51
170	-719,7512849	-719,75128486	-1889707,00
180	-719,7518582	-719,75185816	-1889708,50
190	-719,7536741	-719,75367412	-1889713,27
200	-719,7564269	-719,75642687	-1889720,50
210	-719,7571034	-719,75710337	-1889722,27
220	-719,7560213	-719,75602127	-1889719,43
230	-719,7529993	-719,75299928	-1889711,50
240	-719,7513119	-719,75131193	-1889707,07
250	-719,7509663	-719,75096630	-1889706,16
260	-719,7527893	-719,75278929	-1889710,95
270	-719,7529269	-719,75292688	-1889711,31
280	-719,7533853	-719,75338526	-1889712,51
290	-719,7512849	-719,75128486	-1889707,00
300	-719,7518582	-719,75185816	-1889708,50
310	-719,7536741	-719,75367412	-1889713,27
320	-719,7564269	-719,75642687	-1889720,50
330	-719,7571034	-719,75710337	-1889722,27
340	-719,7560213	-719,75602127	-1889719,43
350	-719,7529993	-719,75299928	-1889711,50

Table S6. Dihedral angles of the model compound *ortho*-methoxyphenylfluorene and their calculated final Gibbs free energy at 293.15 K.

Angle (rel.to staggered) [°]	Energy (abs.) [Eh]	Energy (rel. to staggered) [Eh]	Energy (rel. to ideal conformation) [kJ/mol]
0	-845,5980913	-845,59809129	-2220117,79
10	-845,5995696	-845,59956959	-2220121,67
20	-845,6026820	-845,60268204	-2220129,84
30	-845,6041932	-845,60419317	-2220133,81
40	-845,6052059	-845,60520589	-2220136,47
50	-845,6051642	-845,60516424	-2220136,36
60	-845,6033710	-845,60337096	-2220131,65
70	-845,6027180	-845,60271801	-2220129,94
80	-845,6022865	-845,60228651	-2220128,80
90	-845,6022772	-845,60227724	-2220128,78
100	-845,6027538	-845,60275381	-2220130,03
110	-845,6034294	-845,60342939	-2220131,80
120	-845,6050666	-845,60506658	-2220136,10
130	-845,6050503	-845,60505025	-2220136,06
140	-845,6042092	-845,60420922	-2220133,85
150	-845,6026480	-845,60264797	-2220129,75
160	-845,5994478	-845,59944779	-2220121,35
170	-845,5980254	-845,59802543	-2220117,62
180	-845,5980913	-845,59809129	-2220117,79
190	-845,5995696	-845,59956959	-2220121,67
200	-845,6026820	-845,60268204	-2220129,84
210	-845,6041932	-845,60419317	-2220133,81
220	-845,6052059	-845,60520589	-2220136,47
230	-845,6051642	-845,60516424	-2220136,36
240	-845,6033710	-845,60337096	-2220131,65
250	-845,6027180	-845,60271801	-2220129,94
260	-845,6022865	-845,60228651	-2220128,80
270	-845,6022772	-845,60227724	-2220128,78
280	-845,6027538	-845,60275381	-2220130,03
290	-845,6034294	-845,60342939	-2220131,80
300	-845,6050666	-845,60506658	-2220136,10
310	-845,6050503	-845,60505025	-2220136,06
320	-845,6042092	-845,60420922	-2220133,85
330	-845,6026480	-845,60264797	-2220129,75
340	-845,5994478	-845,59944779	-2220121,35
350	-845,5980254	-845,59802543	-2220117,62

Table S7. Dihedral angles of the model compound *ortho*-methoxyphenylporphyrin and their calculated final Gibbs free energy at 293.15 K.

Angle (rel.to staggered) [°]	Energy (abs.) [Eh]	Energy (rel. to staggered) [Eh]	Energy (rel. to ideal conformation) [kJ/mol]
0	-1333,007508	-1333,00750779	-3499811,21
10	-1333,006239	-1333,00623868	-3499807,88
20	-1333,006225	-1333,00622518	-3499807,84
30	-1333,007424	-1333,00742397	-3499810,99
40	-1333,006484	-1333,00648412	-3499808,52
50	-1333,004215	-1333,00421465	-3499802,57
60	-1332,999541	-1332,99954068	-3499790,29
70	-1332,991515	-1332,99151484	-3499769,22
80	-1332,980299	-1332,98029918	-3499739,78
90	-1332,969251	-1332,96925087	-3499710,77
100	-1332,965505	-1332,96550464	-3499700,93
110	-1332,948099	-1332,94809879	-3499655,23
120	-1332,969695	-1332,96969535	-3499711,94
130	-1332,980937	-1332,98093686	-3499741,45
140	-1332,991737	-1332,99173682	-3499769,81
150	-1332,999709	-1332,99970878	-3499790,74
160	-1333,004283	-1333,00428306	-3499802,75
170	-1333,006467	-1333,00646727	-3499808,48
180	-1333,007508	-1333,00750779	-3499811,21
190	-1333,006239	-1333,00623868	-3499807,88
200	-1333,006225	-1333,00622518	-3499807,84
210	-1333,007424	-1333,00742397	-3499810,99
220	-1333,006484	-1333,00648412	-3499808,52
230	-1333,004215	-1333,00421465	-3499802,57
240	-1332,999541	-1332,99954068	-3499790,29
250	-1332,991515	-1332,99151484	-3499769,22
260	-1332,980299	-1332,98029918	-3499739,78
270	-1332,969251	-1332,96925087	-3499710,77
280	-1332,965505	-1332,96550464	-3499700,93
290	-1332,948099	-1332,94809879	-3499655,23
300	-1332,969695	-1332,96969535	-3499711,94
310	-1332,980937	-1332,98093686	-3499741,45
320	-1332,991737	-1332,99173682	-3499769,81
330	-1332,999709	-1332,99970878	-3499790,74
340	-1333,004283	-1333,00428306	-3499802,75
350	-1333,006467	-1333,00646727	-3499808,48

7. References for Supporting Information

S1. G. M. Sheldrick, A short history of SHELX. *Acta Crystallogr. A: Found. Crystallogr.* **2008**, *64, 112-122*.

S2. G. M. Sheldrick, Crystal structure refinement with SHELXL. *Acta Crystallogr. C: Crystal Struc. Comm.* **2015**, *71, 3-8*.

S3. C. F. Macrae, I. Sovago, S. J. Cottrell, P. T. A. Galek, P. McCabe, E. Pidcock, M. Platings, G. P. Shields, J. S. Stevens, M. Towlera, P. A. Wood, *J. Appl. Crystallogr.* **2020**, *53, 226-235*.

S4. M. Kumada, K. Tamao, K. Sumitani, *Org. Syn.*, **2003**, *58, 127-127*.

S5. S. Y. Chana, Y. C. Loha, C. W. Oo, M. F. Yam, *Bioorg. Chem.* **2020**, *104, 104239.*

S6. J.-H. Fournier, T. Maris, J.D. Wuest, *J. Org. Chem.* **2004**, *69, 1762-1775*.

S7. L. Maqueira, A. Iribarren, A. C. Valdés, C. P. de Meloc, C. G. dos Santos, *J. Porphyrins Phthalocyanines* **2012**, *16*, 267-272.

S8. S. Singto, S. Tantayanon, C. A. Zoto, R. E. Connors, *J. Mol. Struc.* **2018**, *1154*, 114-130.

S9. S. Y. Chan, Y. C. Loh, C. W. Oo, M. F. Yam, *Bioorg. Chem.* **2020**, *104*, 104239.

S10. G. Zheng, J. Shen, Y. Zhan, H. Yi, S. Xue, Z. Wang, X. Ji, Z. Li, *Eur. J. Med. Chem.* **2014**, *81*, 277–288.

S11. M. Janghouri, M. Adineh, *J. Photochem. Photobiol. Chem.* **2017**, *341*, 31–38.

S12. E. Chen, M. Qiu, Y. Zhang, L. He, Y. Sun, H. Zheng, X. Wu, J. Zhang, Q. Lin, *Angew. Chem. Int. Ed.* **2022**, *61*, e202111622.

S13. A. Charisiadis, A. Bagaki, E. Fresta, K. T. Weber, G. Charalambidis, C. Stangel, A. G. Hatzidimitriou, P. A. Angaridis, A. G. Coutsolelos, R. D. Costa, *ChemPlusChem* **2018**, *83*, 254–265.

S14. C. Gomes, M. Peixoto, M. Pineiro, *Molecules* **2021**, *26*, 6652.

S15. P. R. Spackman, M. J. Turner, J. J. McKinnon, S. K. Wolff, D. J. Grimwood, D. Jayatilaka, M. A. Spackman, *J. Appl. Cryst.* **2021**, *54*, 1006–1011.
